# Coalescent-Based Analyses of Genomic Sequence Data Provide a Robust Resolution of Phylogenetic Relationships among Major Groups of Gibbons

**DOI:** 10.1093/molbev/msx277

**Published:** 2017-10-25

**Authors:** Cheng-Min Shi, Ziheng Yang

**Affiliations:** 1 CAS Key Laboratory of Genomic and Precision Medicine, Beijing Institute of Genomics, Chinese Academy of Sciences, Beijing, China; 2 Department of Genetics, Evolution and Environment, University College London, London, United Kingdom; 3 Radcliffe Institute for Advanced Studies, Harvard University, Cambridge, MA 02138, USA

**Keywords:** anomaly zone, astral, bpp, coalescent, concatenation, gene tree, gibbon, species tree, SVDquartets

## Abstract

The phylogenetic relationships among extant gibbon species remain unresolved despite numerous efforts using morphological, behavorial, and genetic data and the sequencing of whole genomes. A major challenge in reconstructing the gibbon phylogeny is the radiative speciation process, which resulted in extremely short internal branches in the species phylogeny and extensive incomplete lineage sorting with extensive gene-tree heterogeneity across the genome. Here, we analyze two genomic-scale data sets, with ∼10,000 putative noncoding and exonic loci, respectively, to estimate the species tree for the major groups of gibbons. We used the Bayesian full-likelihood method bpp under the multispecies coalescent model, which naturally accommodates incomplete lineage sorting and uncertainties in the gene trees. For comparison, we included three heuristic coalescent-based methods (mp-est, SVDQuartets, and astral) as well as concatenation. From both data sets, we infer the phylogeny for the four extant gibbon genera to be (*Hylobates*, (*Nomascus*, (*Hoolock*, *Symphalangus*))). We used simulation guided by the real data to evaluate the accuracy of the methods used. Astral, while not as efficient as bpp, performed well in estimation of the species tree even in presence of excessive incomplete lineage sorting. Concatenation, mp-est and SVDQuartets were unreliable when the species tree contains very short internal branches. Likelihood ratio test of gene flow suggests a small amount of migration from *Hylobates moloch* to *H. pileatus*, while cross-genera migration is absent or rare. Our results highlight the utility of coalescent-based methods in addressing challenging species tree problems characterized by short internal branches and rampant gene tree-species tree discordance.

## Introduction

Gibbons (Hylobatidae), known also as lesser apes, are the closest relatives of hominids (humans and great apes), having diverged in the early to middle Miocene (Matsudaira and Ishida 2010; Carbone et al. 2014). They inhabit the tropical forests of Southeast Asia and have a number of characteristics distinct from the great apes, in body plan (such as smaller body sizes), morphology and anatomy (such as coat colors, hair patterns, and limb bones; Ruff and Runestad 1992), behavior (e.g., social structure, monogamy, and territoriality; Mitani 1984), communication (e.g., vocalizations; Marshall and Marshall 1976; Geissmann 2002; Clarke et al. 2006), and genetics (e.g., the number of chromosomes and synteny; Muller et al. 2003; Roberto et al. 2007; Carbone et al. 2014). There are more species of gibbons than the great apes. Unfortunately, many species of gibbons are endangered or critically endangered due to habitat loss and anthropogenic disturbances (Mittermeier et al. 2013).

Extant gibbons are classified into four genera: *Hylobates*, *Hoolock*, *Nomascus*, and *Symphalangus*, with up to 20 species recognized (9, 3, 7, and 1 in the four genera, respectively), including the newly described skywalker hoolock gibbon (Fan et al. 2017; Anandam et al. 2013). Knowledge of the phylogenetic relationships of the gibbons is important to our understanding of their morphological and behavioral adaptations and to developing good conservation practices. However, the phylogeny of extant gibbon species, and in particular, the relationships among the four extant genera, are unresolved, with previous analyses providing conflicting estimates (Muller et al. 2003; Takacs et al. 2005; Chatterjee et al. 2009; Wall et al. 2013). For example, morphological and anatomical data suggested that *Hylobates* and *Hoolock* were closely related, vocal data grouped *Hylobates* and *Nomascus* (Geissmann 2002), whereas chromosome rearrangement data grouped *Nomascus* and *Symphalangus* (Muller et al. 2003). Different autosomal regions, mitochondrial genomes, Y chromosomal DNA and *Alu* elements also suggested conflicting phylogenies (Matsudaira and Ishida 2010; Chan et al. 2012; Meyer et al. 2012). Some of those differences may be due to estimation artefacts such as homoplasy in morphological characters or systematic errors in phylogenetic reconstruction. However, a major factor is the quick succession of the speciation events (Thinh et al. 2010; Wall et al. 2013). The radiative divergences combined with relatively large population sizes of the ancestral species mean that the stochastic nature of the coalescent process in the ancestral species will cause different regions of the genomes to have different genealogical histories, which may conflict with the species phylogeny.

To study the phylogenetic relationships of the gibbon genera, Carbone et al. (2014) applied the UPGMA method to 100-kb nonoverlapping sliding windows along the genome. This phylogenetic analysis did not account for the coalescent process, and produced a “forest” of phylogenetic trees. All 15 possible rooted trees for the four genera were found in substantial proportions of the sliding windows (Carbone et al. 2014, fig. 5), with frequency 15.4% for the most common tree 1 to 2.8% for the least common tree 15 (table 1). Tree 1, with the topology (*Hylobates*, (*Nomascus*, (*Hoolock*, *Symphalangus*))), was also the Neighbor-Joining tree in the analysis of a coding data set of ∼11,000 exonic regions and another nongenic data set of ∼12,000 noncoding regions, although the support was weak (Carbone et al. 2014). The same coding and noncoding data were analyzed by Veeramah et al. (2015) using a coalescent-based ABC (for Approximate Bayesian Computation) approach. This effort similarly failed to produce a species tree with any confidence. However, as the authors discussed, the ABC approach is based on summary statistics and may lack power.

**Table 1.** Species Tree Numbering According to the Frequency of UPGMA Trees for 100 kb Nonoverlapping Sliding Windows of Carbone et al. (2014), supplementary table ST 8.4, Supplementary Material online).

No.	Topology	Frequency
1	(((S, B), N), H)	0.154
2	(((S, B), H), N)	0.132
3	(((N, B), S), H)	0.109
4	(((N, S), B), H)	0.079
5	(((N, B), H), S)	0.072
6	(((H, B), S), N)	0.067
7	((H, N), (S, B))	0.056
8	(((H, B), N), S)	0.052
9	(((H, S), B), N)	0.051
10	(((N, S), H), B)	0.047
11	(((H, N), B), S)	0.041
12	(((H, S), N), B)	0.038
13	(((H, N), S), B)	0.037
14	((H, S), (N, B))	0.035
15	((H, B), (N, S))	0.028

Note.—Eight sliding windows produced unique trees that fail to recover the H clade (Hm, Hp); these are not listed here.

In the past few years, the multispecies coalescent (MSC) model (Rannala and Yang 2003) has emerged as a powerful framework for inferring species trees while accounting for incomplete lineage sorting due to ancestral polymorphism (e.g., Edwards et al. 2007, 2016; Xu and Yang 2016). The MSC accounts for the coalescent process in both modern and ancestral species and the resulting gene tree-species tree discordance, avoiding the assumption that the same tree underlies all gene loci as in traditional phylogenetic analysis. The method averages over all possible gene trees at each locus, and accommodates the uncertainties in the gene tree at the locus due to limited amount of sequence data through calculation of the sequence likelihood (the probability of the sequence alignment given the gene tree and branch lengths). By combining information at many loci, reliable estimation of the species tree is possible even if every locus has only weak phylogenetic information (Xu and Yang 2016). However, full-likelihood implementations of the MSC (Liu 2008; Heled and Drummond 2010; Yang and Rannala 2014) involve intensive computation and have been impractical for large data sets with a few hundred loci. Recent developments of smart Markov chain Monte Carlo (MCMC) proposals based on the subtree pruning and regrafting (SPR) and node-slider algorithms, which make coordinated changes to both the species tree and the gene trees in the same MCMC move, have improved the mixing efficiency of the algorithm and pushed the limit of Bayesian species tree estimation under the MSC to thousands of loci (Rannala and Yang 2017).

Here, we apply the new algorithms implemented in bpp (Rannala and Yang 2017) to the two genome-scale data sets of Carbone et al. (2014), each consisting of over 10,000 loci. The monophyly of each of the four extant gibbon genera is well supported (Ross 2016), and our objective in this study is to resolve the relationships among the genera rather than among different species of each genus. We include three heuristic coalescent-based methods: mp-est (Liu et al. 2010), SVDQuartets (Chifman and Kubatko 2014), and astral (Mirarab and Warnow 2015), as well as the simple and commonly used method of concatenation (Springer and Gatesy 2016; Edwards et al. 2016). To evaluate the reliability of the methods, we simulated data sets based on parameter estimates obtained from the real data. We also use a recently developed likelihood method (Zhu and Yang 2012; Dalquen et al. 2017) to test for potential gene flow between the gibbon species and assess its impact on estimation of the gibbon phylogeny. Our analyses led to a confident resolution of the phylogenetic relationships among the four extant genera of gibbons, and highlight important differences in statistical performance among competing methods of species tree estimation.

## Results

### Bayesian bpp Analyses of Real and Simulated Data Sets

#### Estimation of the Species Tree Topology Using the Two Full Gibbon Data Sets

We used the Bayesian MCMC program bpp to analyze two genome-scale data sets generated by Carbone et al. (2014) and Veeramah et al. (2015) for five gibbon species: *Hylobates moloch* (Hm), *Hylobates pileatus* (Hp), *Nomascus leucogenys* (N), *Hoolock leuconedys* (B), and *Symphalangus syndactylus* (S). The first data set (Noncoding) includes 12,413 loci, each of 1,000 bp in length. The second data set (Coding) consists of 11,323 coding loci, each of 200 bp. The MSC model (Rannala and Yang 2003) implemented in the bpp program assumes free recombination among loci and no recombination within each locus. The ideal loci should thus be loosely linked short genomic segments that are far apart from each other (Takahata 1986; Burgess and Yang 2008; Lohse et al. 2011). The two gibbon data sets largely fit this description (Carbone et al. 2014, SI text S8.3; Veeramah et al. 2015). Each of the two data sets was analyzed first in full and then as divided subsets.

**Fig. 1.**
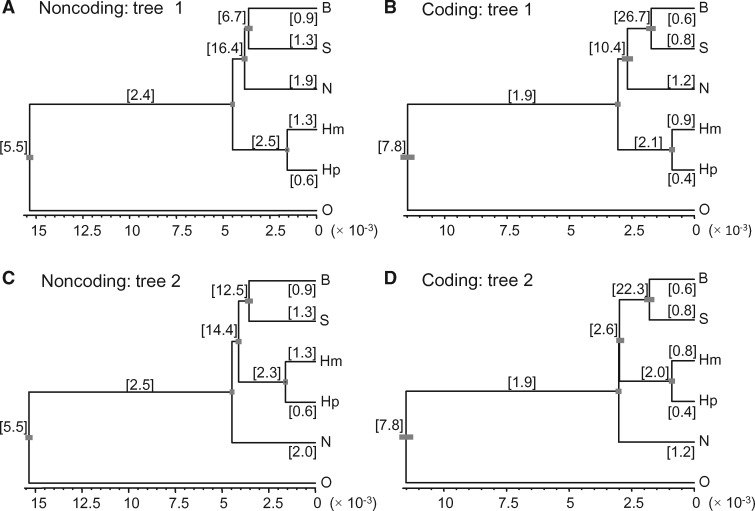
Two species trees obtained in the bpp analysis of the two full real data sets: Noncoding and Coding. Branch lengths are drawn to represent the posterior means of the divergence times (*τ*s) estimated from the A00 analysis with the species tree fixed, and the node bars represent the 95% HPD intervals. The posterior means of *θ*s (×10^−3^) are shown in brackets next to the branches. Species tree 1 is the MAP tree in both data sets according to the marginal likelihood calculation (fig. 2).

In the analysis of the full data sets, we conducted ten independent runs, using the top ten trees of table 1 as starting species trees. All 10 runs converged to either trees 1 or 2 (fig. 1), irrespective of the starting tree. Throughout this paper, we label species tree topologies according to the frequencies of UPGMA trees in the sliding-window analysis of Carbone et al. (2014, supplementary table ST 8.4, Supplementary Material online): for example, trees 1 and 2 are the two most frequent UPGMA gene trees, found in 15.4% and 13.2% of the sliding windows, respectively (table 1). We found that in both data sets, bpp converged to tree 1 in seven runs but to tree 2 in the other three. Additional runs using random starting trees also converged to either trees 1 or 2. However, the Markov chain failed to move between species trees 1 and 2. Note that those two species trees differ by a simple Nearest Neighbor Interchange (NNI) move. The SPR and NodeSlider moves implemented by Rannala and Yang (2017) appear to be effective in moving the chain out of poor species trees in the early stage of the MCMC, but not effective in moving between good species trees after the gene trees at the multiple loci are nearly optimized (see Discussion).

**Fig. 2.**
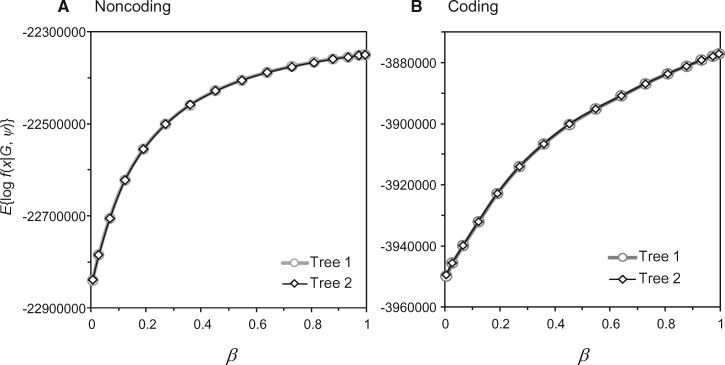
Calculation of the marginal likelihood for species trees 1 and 2 for (*A*) the Noncoding and (*B*) the Coding data sets. The average log likelihood over the MCMC sample from the power posterior is plotted against *β* for each species tree (Rannala and Yang 2017). The log marginal likelihood ratio (or log Bayes factor) for trees 1 against 2 is ≈112 for Noncoding and ≈9 for Coding.

As it is very inefficient to combine the MCMC samples across the multiple runs to estimate posterior probabilities for species trees 1 and 2 (*P*_1_ and *P*_2_), we used the path sampling or thermodynamic integration method (Lartillot and Philippe 2006) to calculate their marginal likelihood values (*M*_1_ and *M*_2_). The procedure is described in Rannala and Yang (2017). The BFdriver program in bpp 3.3 was used to generate the control files for *K* = 16 independent MCMC runs to sample from the different power posterior distributions at different *β* values. The logarithm of the marginal likelihood was given by numerical integration as a sum over the *K* quadrature points (fig. 2). We found that $\text{log}\;\;M_{1}/M_{2}\approx 112$ for Noncoding and ≈9 for Coding, so that $P_{1}/P_{2}$ ≈ e^112^ and e^9^, respectively. Thus species tree 1 was the MAP tree for both data sets, with the posterior ∼1.000.

#### Estimation of Parameters in the MSC Model from the Two Real Data Sets

We estimated the species divergence times (*τ*s) and population sizes (*θ*s) for species trees 1 and 2 for the two full data sets (fig. 1, supplementary table S1, Supplementary Material online). Ten independent runs generated very similar estimates, and the MCMC samples were merged to produce the posterior summary. The posterior credibility intervals (CIs) for *τ*s were very narrow (fig. 1*A* and *B*), due to the huge data size. The parameter estimates on species tree 1 are largely consistent between the coding and noncoding data sets. The posterior means of *τ*s are nearly proportional; fitting the regression line *y *=* bx* to the five points (five pairs of *τ* estimates) led to *τ*_(C)_ = 0.73*τ*_(NC)_, with *r^2^* = 0.985, indicating that the mutation rate for the coding loci was ∼0.73 times that for the noncoding loci. The imperfect correlation was mainly caused by the SB node appearing too young (or *τ*_SB_ too small) in the Coding tree; *τ*_SB_ appeared to be poorly estimated with large sampling errors because the branch is very short. Similarly the posterior means of *θ*s for the extant species are nearly proportional between the two data sets, with the regression line (for five points for S, B, N, Hm, and Hp) to be *θ*_(C)_ = 0.62*θ*_(NC)_, with *r^2^* = 0.986. The correlation was much weaker (*r*^2^ = 0.28) if all 10 estimates of *θ*s on the species tree were used: the estimate ${\hat{\theta }}_{\text{SB}}$ = 0.0267 from the coding loci was too high with a large sampling error; removing that point gave *r*^2^ = 0.84. There is only one sequence for the outgroup (human) so that no *θ* estimate was available.

According to our estimates, the population size for *H. moloch* (Hm) is about twice as large as for *H. pileatus* (Hp), and that for *S. syndactylus* (S) is 1.3 times as large as for *H. leuconedys* (B) (fig. 1*A* and *B*). Population sizes for ancestral species SB and SBN were a few times larger than those for the extant species, although the estimates involve large sampling errors because the branches that represent those ancestral species are very short.

#### Analysis of the Data Subsets

**Fig. 3.**
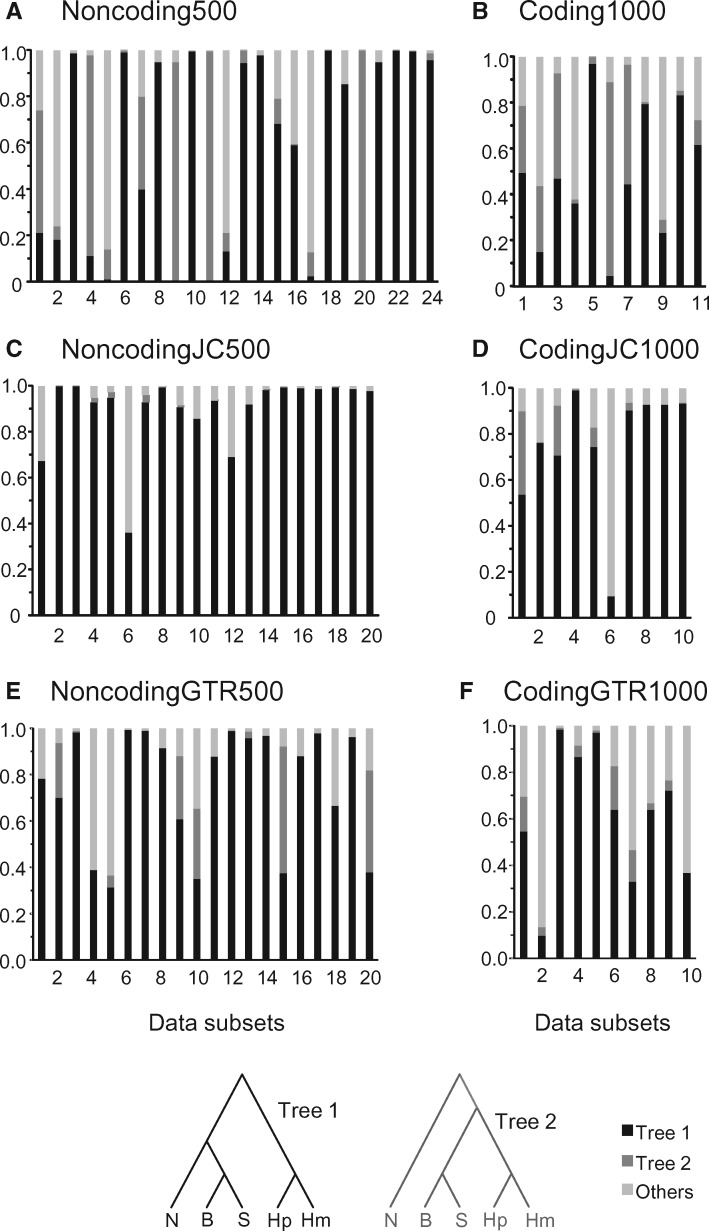
Posterior probabilities for species trees in the bpp analysis of the real (*A* and *B*) and simulated (*C*–*F*) data subsets. The data of (*C*) and (*D*) were simulated under JC and those of (*E*) and (*F*) under GTR+Γ, using species tree 1 and posterior means of parameters (*τ*s and *θ*s) estimated from the real data (fig. 1*A* and *B*).

We separated the noncoding loci into 24 smaller subsets according to their genomic locations in *N. leucogenys*, with each subset consisting of 500 loci (or 913 for the last subset). Similarly, the coding loci were separated into 11 data subsets, each of 1,000 loci (1,323 for the last). Those are referred to as the Noncoding500 and Coding1000 data sets, respectively. The subsets are small enough so that bpp does not suffer from mixing problems but large and informative enough for the species tree to be estimated with confidence. Furthermore, analysis of the subsets is useful for assessing potential heterogeneity across the genome in the evolutionary history among the gibbon species. The bpp runs were successful in the analysis of all data subsets, with no obvious signs of mixing problems encountered. Irrespective of the starting species trees, bpp visited the same set of species trees with substantial posterior probabilities among the 10 replicate runs, although the frequencies with which those species trees were visited may vary somewhat among the runs. The MCMC samples for the runs were then merged to produce the posterior summary (fig. 3*A* and *B*, table 2).

**Table 2.** Species Trees Inferred by Different Methods from Data Subsets for the Two Real and Four Simulated Data Sets.

	Real					JC Data					GTR Data				
Subset	bpp	astral	SVD	ConJC	ConGTR	bpp	astral	SVD	ConJC	ConGTR	bpp	astral	SVD	ConJC	ConGTR
N01	2 (0.53)	2 (0.44)	2 (0.59)	7 (0.78)	7 (0.98)	1 (0.67)	3 (0.44)	1 (0.95)	1 (0.57)	7 (0.53)	1 (0.78)	1 (0.59)	4 (0.98)	1 (0.46)	15 (0.49)
N02	9 (0.25)	9 (0.49)	2 (0.34)	14 (0.60)	14 (0.69)	1 (1.00)	1 (0.87)	1 (1.00)	1 (0.96)	7 (0.66)	1 (0.70)	1 (0.64)	4 (0.51)	1 (0.84)	7 (0.91)
N03	1 (0.99)	1 (0.54)	1 (0.63)	7 (0.68)	7 (0.94)	1 (1.00)	1 (0.95)	1 (0.91)	1 (0.68)	7 (0.93)	1 (0.98)	1 (0.89)	4 (0.56)	1 (0.53)	7 (0.92)
N04	2 (0.87)	2 (0.84)	2 (0.70)	2 (0.83)	7 (0.96)	1 (0.93)	7 (0.55)	7 (0.66)	7 (1.00)	7 (1.00)	1 (0.39)	1 (0.56)	4 (1.00)	3 (0.47)	14 (0.4)
N05	9 (0.50)	12 (0.55)	11 (0.30)	7 (0.35)	14 (0.48)	1 (0.95)	1 (0.56)	7 (0.99)	7 (0.68)	7 (0.99)	7 (0.63)	7 (0.50)	7 (0.96)	7 (1.00)	7 (1.00)
N06	1 (0.99)	1 (0.78)	7 (0.88)	1 (0.65)	7 (0.53)	4 (0.40)	4 (0.39)	5 (1.00)	7 (0.92)	14 (0.96)	1 (0.99)	1 (0.91)	1 (0.99)	7 (0.49)	7 (0.98)
N07	2 (0.40)	2 (0.63)	2 (0.60)	7 (0.42)	7 (0.84)	1 (0.93)	1 (0.44)	2 (0.50)	7 (0.82)	7 (0.94)	1 (0.99)	1 (0.69)	4 (1.00)	7 (0.67)	7 (0.83)
N08	1 (0.95)	1 (0.63)	1 (0.37)	7 (0.59)	7 (0.77)	1 (0.99)	1 (0.94)	1 (1.00)	1 (0.63)	7 (0.97)	1 (0.91)	1 (0.80)	1 (1.00)	7 (0.80)	7 (1.00)
N09	2 (0.95)	2 (0.50)	2 (0.57)	2 (0.75)	7 (0.69)	1 (0.91)	1 (0.60)	1 (0.57)	7 (0.96)	7 (0.96)	1 (0.61)	1 (0.61)	1 (1.00)	7 (0.97)	7 (0.98)
N10	1 (1.00)	1 (0.64)	6 (0.39)	2 (0.58)	7 (0.55)	1 (0.86)	1 (0.73)	1 (0.70)	7 (0.86)	7 (0.87)	1 (0.35)	2 (0.37)	7 (0.41)	7 (0.78)	7 (0.91)
N11	2 (0.99)	2 (0.74)	2 (0.62)	2 (0.94)	7 (0.41)	1 (0.94)	1 (0.68)	1 (0.97)	7 (0.86)	7 (0.97)	1 (0.88)	1 (0.69)	7 (0.94)	7 (0.85)	7 (0.91)
N12	14 (0.25)	9 (0.58)	3 (0.24)	14 (0.85)	14 (0.84)	1 (0.69)	1 (0.61)	4 (1.00)	1 (0.41)	7 (0.36)	1 (0.99)	1 (0.88)	1 (0.95)	7 (0.90)	7 (0.97)
N13	1 (0.94)	1 (0.80)	1 (0.43)	1 (0.77)	7 (0.81)	1 (0.92)	1 (0.54)	3 (1.00)	1 (0.60)	1 (0.54)	1 (0.96)	1 (0.73)	2 (0.70)	7 (0.70)	7 (0.93)
N14	1 (0.98)	1 (0.86)	1 (0.38)	7 (0.66)	7 (0.97)	1 (0.98)	1 (0.56)	7 (0.98)	7 (1.00)	7 (1.00)	1 (0.97)	1 (0.59)	1 (0.84)	7 (0.91)	7 (0.91)
N15	1 (0.68)	9 (0.41)	6 (0.33)	2 (0.61)	7 (0.66)	1 (1.00)	1 (0.73)	11 (0.96)	7 (0.54)	7 (1.00)	2 (0.55)	1 (0.48)	7 (0.75)	7 (0.99)	7 (1.00)
N16	1 (0.59)	9 (0.66)	5 (0.35)	14 (0.82)	14 (0.88)	1 (0.99)	1 (0.88)	3 (0.55)	7 (0.63)	7 (0.92)	1 (0.88)	1 (0.66)	6 (1.00)	14 (0.46)	14 (0.51)
N17	9 (0.41)	9 (0.67)	9 (0.46)	14 (0.86)	14 (0.91)	1 (0.99)	1 (0.81)	1 (0.85)	1 (0.85)	7 (0.80)	1 (0.98)	1 (0.84)	1 (1.00)	1 (0.98)	7 (0.58)
N18	1 (1.00)	1 (0.78)	7 (0.72)	1 (0.50)	7 (0.84)	1 (0.99)	1 (0.64)	7 (0.68)	7 (0.97)	7 (1.00)	1 (0.66)	4 (0.41)	3 (0.91)	1 (0.79)	7 (0.71)
N19	1 (0.85)	3 (0.45)	7 (0.55)	7 (0.73)	7 (0.90)	1 (0.99)	1 (0.85)	1 (1.00)	7 (0.55)	7 (0.89)	1 (0.96)	1 (0.83)	1 (1.00)	7 (0.97)	7 (0.98)
N20	2 (1.00)	2 (0.67)	7 (0.57)	2 (0.75)	7 (0.76)	1 (0.98)	1 (0.93)	4 (0.98)	1 (0.64)	7 (0.60)	2 (0.44)	12 (0.39)	6 (1.00)	14 (0.68)	14 (0.69)
N21	1 (0.95)	1 (0.56)	7 (0.53)	1 (0.78)	7 (0.72)										
N22	1 (1.00)	1 (0.91)	7 (0.59)	1 (0.64)	7 (0.88)										
N23	1 (1.00)	1 (0.82)	1 (0.41)	7 (0.98)	7 (0.99)										
N24	1 (0.96)	1 (0.59)	5 (0.48)	15 (0.37)	15 (0.64)										
C01	1 (0.50)	3 (0.42)	1 (0.38)	2 (0.33)	14 (0.44)	1 (0.53)	2 (0.64)	1 (0.72)	1 (0.43)	7 (0.66)	1 (0.55)	13 (0.60)	10 (0.38)	7 (0.27)	7 (0.43)
C02	2 (0.26)	2 (0.54)	8 (0.93)	4 (0.38)	14 (0.32)	1 (0.76)	1 (0.53)	4 (0.72)	4 (0.42)	15 (0.48)	14 (0.24)	5 (0.37)	4 (0.73)	14 (0.82)	14 (0.84)
C03	1 (0.47)	1 (0.56)	7 (0.98)	1 (0.55)	1 (0.58)	1 (0.71)	1 (0.75)	1 (0.89)	1 (0.82)	7 (0.61)	1 (0.98)	1 (0.89)	4 (0.56)	1 (0.66)	7 (0.42)
C04	1 (0.36)	4 (0.38)	14 (0.63)	1 (0.46)	7 (0.41)	1 (0.99)	1 (0.90)	4 (0.99)	1 (0.57)	7 (0.53)	1 (0.87)	7 (0.45)	1 (1.00)	7 (0.89)	7 (0.97)
C05	1 (0.97)	1 (0.76)	4 (0.56)	1 (0.77)	1 (0.84)	1 (0.74)	1 (0.62)	4 (0.32)	14 (0.35)	14 (0.47)	1 (0.97)	1 (0.73)	1 (1.00)	1 (0.70)	7 (0.73)
C06	2 (0.85)	2 (0.53)	7 (0.73)	2 (0.51)	1 (0.57)	3 (0.88)	3 (0.89)	3 (0.69)	3 (0.64)	14 (0.78)	1 (0.64)	1 (0.42)	1 (0.91)	7 (0.85)	7 (0.95)
C07	2 (0.52)	1 (0.71)	4 (0.99)	1 (0.31)	14 (0.58)	1 (0.90)	1 (0.76)	2 (0.98)	7 (0.62)	7 (0.91)	7 (0.53)	7 (0.48)	7 (0.91)	7 (0.98)	7 (1.00)
C08	1 (0.79)	1 (0.52)	7 (1.00)	1 (0.53)	7 (0.44)	1 (0.93)	1 (0.88)	3 (0.85)	1 (0.65)	7 (0.61)	1 (0.64)	1 (0.69)	1 (0.88)	7 (0.47)	7 (0.69)
C09	3 (0.47)	9 (0.48)	9 (0.58)	14 (0.5)	14 (0.72)	1 (0.93)	1 (0.82)	1 (0.98)	1 (0.87)	1 (0.53)	1 (0.72)	1 (0.46)	3 (0.72)	7 (0.66)	7 (0.55)
C10	1 (0.83)	1 (0.59)	7 (0.87)	1 (0.42)	7 (0.41)	1 (0.94)	1 (0.70)	3 (0.98)	1 (0.75)	1 (0.43)	4 (0.54)	4 (0.71)	1 (0.50)	15 (0.44)	15 (0.60)
C11	2 (0.53)	1 (0.51)	4 (0.62)	1 (0.64)	7 (0.56)										

Note.—ConJC and ConGTR are concatenation analyses by PhyML under the JC or GTR+Γ_4_ models, respectively.

Out of the 24 Noncoding500 data subsets (N1–N24 in table 2), four distinct MAP trees were observed: species tree 1 in 14 data sets with posterior ranging from 0.59 to 1.00; tree 2 in six data sets with posterior from 0.40 to 1.00, and trees 9 and 14 in three and one data set, respectively, with posterior ≤0.50 (fig. 3*A* and table 2). Thus the posterior was never very high except when the MAP tree was species trees 1 or 2.

Out of the 11 Coding1000 data subsets (C1–C11 in table 2), three distinct MAP trees were observed: tree 1 in 6 data sets with posterior 0.36–0.97, tree 2 in 4 data sets with posterior 0.26–0.85, and tree 3 only once with posterior 0.47 (fig. 3*B* and table 2). The Coding1000 data sets, even with twice as many loci, are far less informative than the Noncoding500 data sets, because they have shorter sequences (200 sites instead of 1000) and lower mutation rates. While tree 1 was the most commonly favored species tree, the support was not very high. No other species tree received strong support in any of the 11 data sets.

Thus the analyses of the data subsets did not suggest heterogeneous evolutionary histories among different regions of the genome beyond the expectations of the coalescent model, and that overall the whole genome covered by those loci appeared to be consistent with tree 1 (or to a lesser extent with tree 2).

#### The Simulated Data Sets

We used species tree 1 and the parameter estimates under the MSC (the posterior means of *τ*s and *θ*s in the A00 analysis, fig. 1*A* and *B*) to simulate two data sets under JC (Jukes and Cantor 1969) (NoncodingJC and CodingJC) and two data sets under GTR+Γ (Yang 1994a, 1994b) (NoncodingGTR and CodingGTR), with the same taxon sampling scheme as in the real data. The GTR data were simulated with the parameters of the GTR+Γ model varying among loci (see Materials). Each of noncoding data sets includes 10,000 alignments (loci), of 1,000 bp, whereas each of the coding data sets includes 10,000 loci each of 200 bp. The data sets were analyzed in the same way as the real data sets, first in full and then divided as subsets.

In the analysis of the four full data sets, all 10 independent bpp runs converged to the true species tree (tree 1), with posterior ∼1.00, except for NoncodingJC. In that data set, eight out of the ten runs converged to tree 1 (the true tree), but the chain was stuck in a wrong tree in two other runs. Compared with the coding loci, the noncoding loci have longer sequences and higher mutation rate, and should be more informative and should estimate the true species tree with higher accuracy and higher precision (that is, the MAP species tree should be the true tree with higher probability and the MAP species tree should have higher posterior). Thus we did not calculate the marginal likelihood values for this data set and concluded that the Bayesian MSC method inferred the true species tree with full support in all four simulated data sets, but bpp had mixing problems in one of them.

**Fig. 4.**
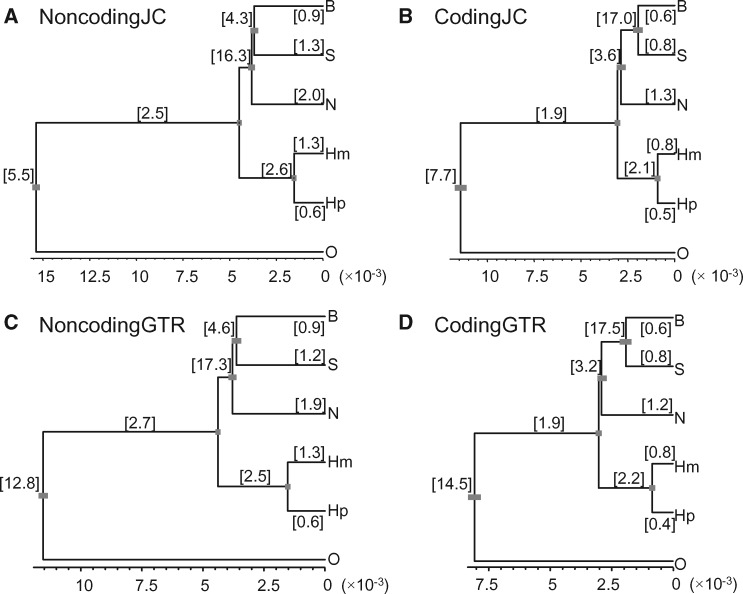
Bpp estimates of parameters (*τ*s and *θ*s) on species tree 1 from the four simulated full data sets. The true parameter values are shown in figure 1*A* and *B*. See legend to figure 1.

The posterior estimates of parameters from the simulated data sets are shown in figure 4. For the two JC data sets, the posterior means were very close to the true values with the exception that in CodingJC, *θ*_SB_, and *θ*_SBN_ for the two very short internal branches were not reliably estimated. For the two GTR data sets, the posterior means of parameters for the nonroot nodes were similarly close to the true values. However, in both data sets, the age of the root (*τ*) is underestimated (0.0115 vs. the true value 0.0153 for NoncodingGTR and 0.0080 vs. 0.0115 for CodingGTR) and the population size parameter for the root (*θ*) is overestimated (0.0128 vs. 0.0055 for NoncodingGTR and 0.0145 vs. 0.0078 for CodingGTR). The heterogeneity in the mutation process among loci in the GTR data is misinterpreted as heterogeneity in the gene trees from the coalescent process, leading to an inflated ancestral population size (*θ*) for the root and a reduced species divergence time (*τ*). This is the same pattern observed in a previous analysis of the hominoid genomics data (Burgess and Yang 2008), in which the parameters for the root species was found to be sensitive to possible heterogeneity in the evolutionary process among loci. We emphasize here that even though the JC model is grossly wrong, the bpp estimates of parameters for the nonroot nodes of the species tree were robust.

The bpp analyses of the simulated data subsets are summarized in table 2 and figure 3. The runs were successful in every case. For the NoncodingJC500 data, bpp inferred the correct species tree 1 in 19 out of the 20 subsets, often with high posterior, while the single wrong tree (tree 4) had very weak support (0.40). For CodingJC1000, bpp inferred the correct tree 1 in 9 out of the 10 subsets, but the posterior was lower than in the noncoding data, and again the single wrong tree had weak support (0.88). As in the real data, the coding subsets are less informative than the noncoding subsets. In the GTR subsets, bpp made 3 errors out of 20 for NoncodingGTR500, and 3 errors out of 10 for CodingGTR1000. The wrong trees all had low support. Overall, the GTR subsets are less informative with lower support for the estimated species trees than the JC subsets. Because the JC and GTR data were simulated using the same values of MSC parameters (*τ*s and *θ*s) so that the gene trees should have similar branch lengths, the GTR data, due to biased base compositions and mutation rates as well as variable mutation rates among sites, should have fewer informative sites than the JC data.

In summary, bpp inferred the correct species tree with 1000 or 500 loci most of the time, and never attached high posteriors to wrong trees in any data sets. This is the case for the GTR data sets as well. Even though JC assumed by bpp is grossly wrong, the analysis is quite robust, with low error rates (table 3).

**Table 3.** Error Rates for bpp, astral, SVDQuartets, and Concatenation (PhyML) in Analysis of Simulated Data Subsets.

	bpp	astral	SVD	ConJC	ConGTR
JC data					
NoncodingJC500 (500 loci, 1,000 sites)	1/20	3/20	11/20	12/20	19/20
CodingJC1000 (1,000 loci, 200 sites)	1/10	2/10	7/10	4/10	8/10
GTR data					
NoncodingGTR500 (500 loci, 1,000 sites)	3/20	4/20	13/20	15/20	20/20
CodingGTR1000 (1,000 loci, 200 sites)	3/10	5/10	5/10	8/10	10/10

Note.—JC was assumed in the analysis of the JC data sets and GTR+Γ in the GTR data sets by astral (using PhyML), whereas JC is assumed in all bpp analyses.

### astral Analyses of Real and Simulated Data Sets

**Fig. 5.**
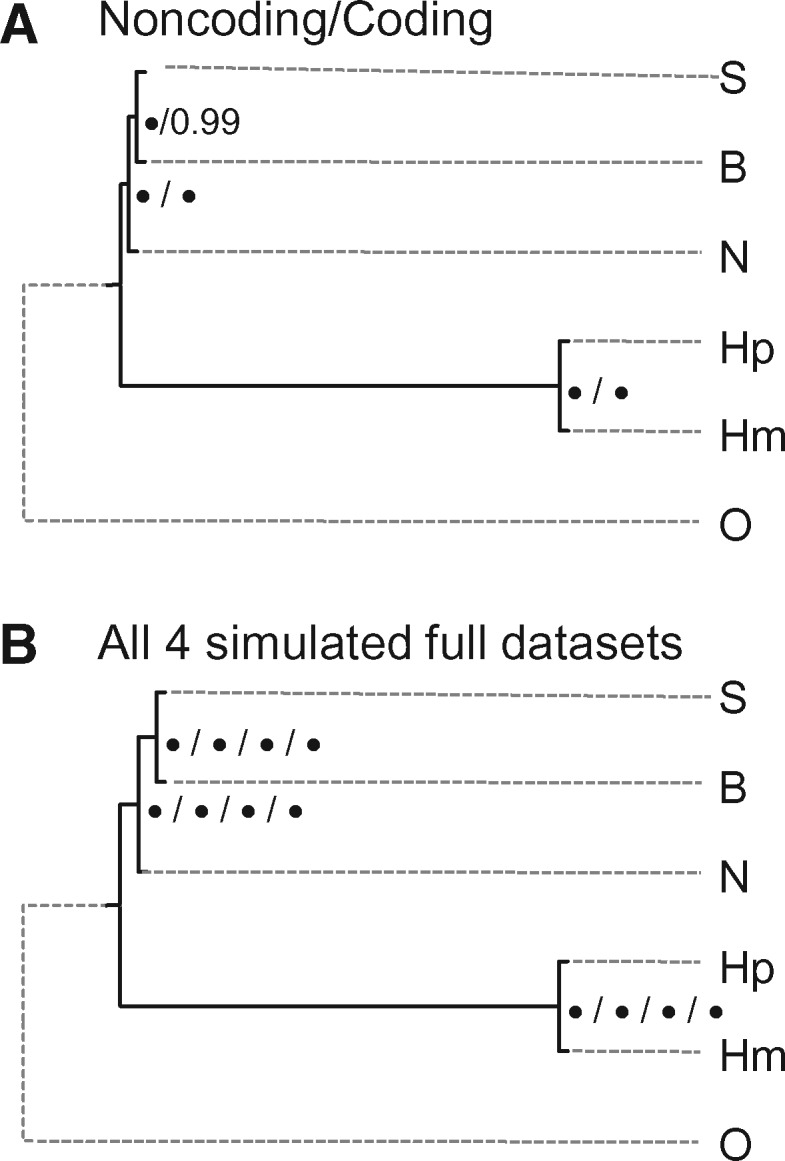
Species trees inferred by astral from the two real (*A*) data sets and four simulated (*B*) data sets. The trees are rooted with human (O) as outgroup. The same tree (tree 1) is inferred in all six data sets. Support values are 100% for all nodes in all analyses (not shown) except that for the Coding data set, the SB node had 99%. Estimates of internal branch lengths are in table 5. Note that astral cannot estimate external branch lengths.

Like bpp, astral inferred species tree 1 in the analyses of two real and four simulated full data sets (table 4 and fig. 5). For the simulated data, the inferred tree was also the true tree. The “local posterior” support value was 100% for every node in every data set except that for the Coding data set, the clade (B, S) had 99% (fig. 5).

**Table 4.** Species Trees (with support values) Inferred by Different Methods from the Real and Simulated Full Data Sets.

	Real Data		Simulated (JC)		Simulated (GTR)	
Method	Noncoding	Coding	Noncoding	Coding	Noncoding	Coding
bpp	1 (1.00)	1 (1.00)	1 (1.00)	1 (1.00)	1 (1.00)	1 (1.00)
astral	1 (1.00)	1 (1.00)	1 (1.00)	1 (1.00)	1 (1.00)	1 (1.00)
SVDQuartets	7 (1.00)	7 (0.96)	1 (1.00)	1 (0.95)	1 (1.00)	1 (0.95)
MP-EST	2	a	3	9	1	9
Concatenation						
PhyML-JC	7 (0.41)	1 (0.95)	7 (1.00)	1 (0.95)	7 (1.00)	7 (0.88)
MrBayes-JC	1 (1.00)	1 (1.00)	7 (1.00)	1 (1.00)	7 (1.00)	7 (1.00)
PhyML-GTR+Γ_4_	7 (1.00)	7 (0.70)	7 (1.00)	7 (0.96)	7 (1.00)	7 (1.00)
MrBayes-GTR+Γ_4_	7 (1.00)	7 (1.00)	7 (1.00)	7 (1.00)	7 (1.00)	7 (1.00)

Note.—The trees are identified in table 1. For the four simulated data sets, tree 1 is the true tree. For bpp, support value is the posterior probability for the MAP tree. For astral, SVDQuartets, and concatenation, it is the minimum support value among the internal nodes, which may be an overestimate of the support for the whole tree. The H node (Hm and Hp) had full support except stated otherwise.

a MP-EST estimated (Hp, ((S, B), (N, Hm))) in the real Coding data, failing to recover the H clade.

As astral and bpp produced the same species tree (tree 1) in all six full data sets, we compared the parameter estimates in the MSC model by the two methods. Astral makes use of gene tree topologies but not gene-tree branch lengths, and is thus unable to identify or estimate most of the parameters in the model. For example, there are 15 parameters (10 *θ*s and 5 *τ*s) on species tree 1 (fig. 1*A*), and astral can estimate only three. The gene tree-species tree topological mismatches around an internal branch provide information about the time length of the branch relative to the ancestral population size for the branch, and as a result, astral can estimate the internal branch lengths on the species tree in coalescent units. This branch length is 2Δ*τ*/*θ* in bpp, where Δ*τ* is the difference in species divergence times for the branch and *θ* is the population size parameter for the branch, and its posterior can be generated using the MCMC samples from the A00 analysis: *τ_i_* and *θ_i_* (the sampled parameter values at MCMC iteration *i*).

The estimates of internal branch lengths (2Δ*τ*/*θ*) obtained by astral and bpp were comparable (table 5). For the real data (Noncoding and Coding), astral estimated a shorter SB branch (meaning that there was more gene-tree conflicts around the branch) than bpp. For the simulated data sets, the astral estimates are close to but often slightly larger than the true values. Note that phylogenetic reconstruction errors inflate the gene tree-species tree conflicts, so that use of reconstructed instead of true gene trees should lead to underestimation of branch lengths (Yang 2002; Huang and Knowles 2009). Here, astral uses the correction of Sayyari and Mirarab (2016) to account for gene-tree estimation errors, which appeared to be effective in this simulation. The bpp estimates were close to the true values in the informative noncoding data sets (NoncodingJC and NoncodingGTR) but were somewhat too large for the short SB branch in the less informative coding data sets (CodingJC and CodingGTR). This appears to be due to the impact of the prior, which specifies too large Δ*τ* and too small *θ* for the short SB branch, relative to the posterior. Nevertheless, the internal branch lengths in coalescent units (2Δ*τ*/*θ*) were better estimated than the *τ* and *θ* parameters for those short branches (compare table 5 with fig. 4). The simple JC model assumed by bpp is seen to produce good parameter estimates in the two GTR data sets as well. We suggest that bpp instead of astral be used for parameter estimation as bpp can estimates all parameters in the model and can provide CIs to indicate the sampling errors.

**Table 5.** astral and bpp Estimates of Internal Branch Lengths in Coalescent Units (2Δ*τ*/*θ*) in Species Tree 1 from the Two Real and Four Simulated Full Data Sets.

Method	SB	SBN	HpHm	SB	SBN	HpHm
	Noncoding			Coding		
astral (JC)	0.039	0.042	2.254	0.044	0.073	2.136
bpp	0.065	0.078	2.330	0.072	0.075	2.051
	(0.055, 0.076)	(0.070, 0.087)	(2.275, 2.386)	(0.057, 0.088)	(0.056, 0.095)	(1.969, 2.137)
bpp_a_	0.065	0.078	2.330	0.071	0.074	2.050
	NoncodingJC			CodingJC		
Truth	0.065	0.078	2.330	0.071	0.074	2.050
astral (JC)	0.072	0.076	2.541	0.083	0.084	2.295
bpp	0.064	0.081	2.264	0.109	0.099	2.062
	(0.052, 0.076)	(0.071, 0.092)	(2.207, 2.323)	(0.089, 0.128)	(0.073, 0.126)	(1.974, 2.155)
bpp_a_	0.064	0.081	2.263	0.108	0.099	2.062
	NoncodingGTR			CodingGTR		
Truth	0.065	0.078	2.330	0.071	0.074	2.050
astral (GTR+Γ_4_)	0.089	0.064	2.581	0.089	0051	2.116
bpp	0.070	0.069	2.318	0.114	0.073	1.969
	(0.057, 0.082)	(0.059, 0.079)	(2.258, 2.379)	(0.094, 0.134)	(0.046, 0.099)	(1.887, 2.054)
bpp_a_	0.070	0.068	2.318	0.113	0.073	1.969

Notes.—In the bpp analysis, the posterior means and 95% equal-tail CIs (in parentheses) are calculated by averaging 2Δ*τ*/*θ* over the MCMC sample from the A00 analysis. The approximate method (bpp_a_) simply uses the posterior means of *τ*s and *θ*s.

The astral analyses of the real data subsets are summarized in table 2. Among the 24 Noncoding500 subsets, tree 1 was the inferred tree 11 times, with 6 times for tree 2, and 5 times for tree 9. Among the 11 Coding1000 subsets, tree 1 was the inferred tree 6 times, with tree 2 twice. Support is low (<95% in every subset), especially in the coding subsets. In general, astral showed more variation among subsets than bpp.

**Fig. 6.**
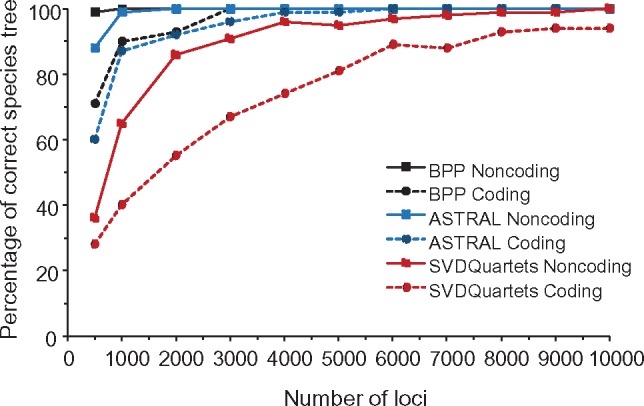
The percentage of correct species trees in simulated data sets by three different methods, plotted against the number of loci. The data were simulated under JC using parameter estimates obtained from the bpp (A00) analysis of the Coding and Noncoding gibbon data sets. For astral and SVDQuartets, the number of replicates is 100, while for bpp, it is 100 for the 500-loci data sets and 30 for others.

In the analysis of the simulated data subsets, astral made more errors than bpp (tables 2 and 3). This was the case even for the GTR data sets, in which case the true GTR+Γ model was assumed in astral whereas the wrong JC model was used in bpp. The astral support value for the estimated species tree never exceeded 95% in any of the subsets, in contrast to bpp, which inferred the true species tree with high posterior in many subsets (table 2). This suggests either that the subsets may be too small for astral to infer the species tree with confidence, or that the astral support values may be too conservative. Figure 6 suggests that the former is the case, because at such data sizes (500 noncoding or 1,000 coding loci), astral does not recover the species tree with very high frequency.

### SVDQuartets Analyses of Real and Simulated Data Sets

The species trees inferred by SVDQuartets in the analyses of the full data sets are shown in table 4. In both real data sets (Noncoding and Coding), SVDQuartets inferred species tree 7, with the topology ((N, H), (B, S)), where H stands for *Hylobates* or the (Hm, Hp) clade. The support value was 100% for every node for Noncoding whereas for Coding, the two nodes had slightly weaker support (96% and 97%).

For the four simulated data sets, the inferred species tree was tree 1 (the true tree; table 4), again with high support. This result may be expected because SVDQuartets estimation of the species tree is consistent since the species tree is identifiable in the case of quartets (four species and four sequences; Chifman and Kubatko 2015) and the quartets uniquely determine the species tree (e.g., Allman et al. 2011). Note that SVDQuartets was developed under the GTR+Γ+I model and includes GTR+Γ and JC as special cases. Thus whereas both bpp and astral estimated species tree 1 in all the six full data sets (two real and four simulated), SVDQuartets recovered species tree 1 (the true tree) in the four simulated data sets, but favored tree 7 in the two real data sets. We discuss this discrepancy later in Discussion.

The SVDQuartets analyses of the real data subsets produced highly variable results (table 2). Out of the 24 Noncoding500 data subsets, tree 7 was the best supported tree 6 times, with 6 times for tree 2, and 5 times for tree 1. Out of the 11 Coding1000 subsets, tree 7 was the best tree 4 times (once for tree 1). Support was high more often in the coding subsets (>95% in 3 out of the 11 subsets) than in the noncoding subsets (none at 95%). This is somewhat surprising, as the noncoding subsets are much more informative, with stronger support for both bpp and astral species trees than the coding subsets.

The analysis of the simulated data subsets by SVDQuartets is summarized in table 2. The method made many errors: 11 and 7 for the JC data sets, compared with 1 and 1 for bpp; and 13 and 5 for the GTR data sets, compared with 3 and 3 for bpp (table 3). A large proportion of the erroneous species trees inferred by SVDQuartets were tree 7. The support value was not related to the correctness of the inferred species tree. High support (>95%) was attached to 7 out of the 11 wrong species trees for NoncodingJC500, to 6 out of the 13 wrong trees for NoncodingGTR500, and to 3 out of the 7 wrong trees for CodingJC1000. Those results suggest that the SVDQuartets support value is unreliable and overconfident, and that the strong support for the conflicting species trees among the Coding1000 real data subsets (table 2) is spurious.

### mp-est Analyses of Real and Simulated Data Sets

In the analysis of the two real full data sets, mp-est inferred tree 2 for Noncoding, and the tree (Hp, ((S, B), (N, Hm))) for Coding. This latter tree fails to group the two *Hylobates* species into the same clade and should be wrong.

When mp-est was applied to the four simulated full data sets, it inferred the correct species tree (tree 1) in only one of them: NoncodingGTR. The inferred incorrect tree was tree 9 for CodingJC and CodingGTR, and tree 3 for NoncodingJC (table 4). This poor performance may be due to the fact that many of the loci had few parsimony-informative sites with very weak phylogenetic information so that the reconstructed gene trees had large uncertainties and errors. Gene-tree errors are known to have a considerable adverse effect on mp-est (Liu et al. 2015; Xu and Yang 2016). Because mp-est performed poorly in the simulated full data sets, we did not examine its performance in the data subsets or consider its results for the real data sets any further.

### Concatenation Analyses of Real and Simulated Data Sets

We used maximum likelihood (ML) implemented in PhyML (Guindon 2013) and Bayesian inference (BI) implemented in MrBayes (Ronquist et al. 2012) to analyze the concatenated alignments for the full data sets, under the JC and GTR+Γ_4_ models. In all analyses, the sequences from the same species formed monophyletic groups, making it possible to extract a species phylogeny from the estimated gene trees. The results are summarized in table 4. Under JC, MrBayes recovered species tree 1 in both real data sets, with full support for all nodes, while PhyML inferred tree 7 from the Noncoding data set, although the bootstrap support for the node (H, N) is only 41%. Under GTR+Γ_4_, both PhyML and MrBayes recovered tree 7 from both the Noncoding and Coding data sets. Note that tree 7 is the balanced tree: ((H, N), (S, B)) (table 1).

In the analysis of the data sets simulated under JC (NoncodingJC and CodingJC), the true species tree (tree 1) was recovered only from CodingJC under the JC model, while the incorrect tree 7 was inferred in the other three data-model combinations (table 4). In particular, under GTR+Γ_4_, both PhyML and MrBayes inferred tree 7. Support values were always high, with bootstrap values >95% and Bayesian posterior ∼100% for all nodes in all analyses. In the analysis of the GTR data sets (NoncodingGTR and CodingGTR), both ML and BI under both JC and GTR+Γ_4_ inferred the incorrect species tree 7, with high support.

We also applied ML (PhyML) to the concatenated alignments of the real data subsets (table 2). In the JC analysis of the 24 Noncoding500 subsets, tree 1 was the ML tree 5 times, with 8 times for tree 7. In the JC analysis of the 11 Coding1000 subsets, tree 1 was recovered 7 times, whereas tree 7 was never the ML tree. However, when GTR+Γ_4_ was assumed, tree 7 was the most common ML tree in both the coding and noncoding subsets.

The ML analysis of the simulated JC data subsets was summarized in table 2. PhyML/JC recovered the true tree 1 in only 8 out of the 20 NoncodingJC500 subsets and 6 out of the 10 CodingJC1000 subsets, whereas tree 7 were inferred from 12 NoncodingJC500 subsets and from one CodingJC1000 subset. When GTR+Γ_4_ model was used, the inferred tree was predominantly tree 7. In the analysis of the GTR data subsets, the inferred species tree was predominantly tree 7, whether JC or GTR+Γ_4_ was assumed by PhyML. It appears that in those simulated data sets, the concatenation/ML method of species tree estimation is inconsistent (see Discussion).

### Test of Migration and Estimation of Migration Rates Using Triplets

We used the ML program 3s to test for gene flow (migration) between the gibbon species and to estimate the directions and rates of migration (Zhu and Yang 2012; Dalquen et al. 2017). The program works with three species only, with three sequences per locus. Thus we constructed triplet data sets by sampling three sequences per locus from each of the Noncoding and Coding data sets (table 6). We fitted two models using ML. Model M0 (no gene flow) assumes no migration and fits the MSC model to the species tree ((*A*, *B*), *C*). Model M2 (gene flow) allows migration between *A* and *B*, with two additional parameters: *M_AB_* and *M_BA_*, where *M_AB_* is the expected number of immigrants in population *B* from population *A* per generation, and so on. MLEs of parameters and the log likelihood values under models M0 and M2 for the triplet data sets are summarized in table 6. We use the likelihood ratio test (LRT) to compare models M0 and M2.

**Table 6.** Estimates of Parameters (×10^−3^) under the MSC Model with Migration for Three Species.

Data & Model	*τ_ABC_*	*τ_AB_*	*θ_ABC_*	*θ_AB_*	*θ*_A_	*θ*_B_	*M_AB_*	*M_BA_*	ℓ	2Δℓ
Noncoding										
Hm-B-O M0	15.17	4.23	5.60	2.53	1.36	0.90			−2238959.32	
Hm-B-O M2	15.17	4.36	5.60	2.40	1.36	0.85	1.12	0.15	−2238928.59	61.46
Hm-S-O M0	15.23	4.28	5.61	2.54	1.43	1.22			−2253001.66	
Hm-S-O M2	15.23	4.38	5.61	2.44	1.39	1.22	0.16	1.20	−2252990.65	22.03
Hm-N-O M0	15.10	4.28	5.70	2.43	1.33	1.88			−2253031.08	
Hm-N-O M2	15.10	4.37	5.70	2.33	1.29	1.89	0.00	1.36	−2253019.96	22.23
B-S-O M0	15.21	4.22	5.63	2.40	0.91	1.22			−2236902.43	
B-S-O M2	15.20	4.34	5.63	2.27	0.87	1.22	0.04	1.10	−2236881.40	42.07
B-N-O M0	15.12	4.21	5.62	2.38	0.92	1.78			−2238554.36	
B-N-O M2	15.12	4.25	5.62	2.34	0.90	1.78	0.00	0.55	−2238549.60	9.52
S-N-O M0	15.17	4.30	5.67	2.33	1.20	1.83			−2252698.72	
S-N-O M2	15.17	4.33	5.66	2.30	1.20	1.82	0.44	0.00	−2252697.60	2.24
Hm-Hp-O M0	15.11	1.42	5.82	2.76	1.26	0.61			−2110388.99	
Hm-Hp-O M2	15.11	1.66	5.82	2.55	1.29	0.48	8.39	0.00	−2110332.65	112.69
Hm-Hp-B M0	4.32	1.49	2.45	2.60	1.34	0.59			−945232.51	
Hm-Hp-B M2	4.31	1.74	2.47	2.32	1.36	0.49	7.82	0.00	−945188.92	87.18
Coding										
Hm-B-O M0	11.27	2.76	8.17	2.29	0.94	0.56			−353419.45	
Hm-B-O M2	11.27	2.91	8.17	2.14	0.91	0.53	1.15	1.05	−353416.45	6.01
Hm-S-O M0	11.34	2.76	8.22	2.40	0.96	0.77			−356126.75	
Hm-S-O M2	11.34	2.87	8.22	2.31	0.93	0.75	0.83	1.10	−356126.18	1.14
Hm-N-O M0	11.26	2.68	8.21	2.61	1.00	1.22			−356688.67	
Hm-N-O M2	11.26	2.85	8.21	2.45	0.98	1.14	3.57	0.72	−356687.58	2.19
B-S-O M0	11.31	2.52	8.21	2.49	0.58	0.81			−352745.31	
B-S-O M2	11.31	2.80	8.22	2.24	0.52	0.77	1.82	2.26	−352740.97	8.69
B-N-O M0	11.32	2.71	8.03	2.22	0.55	1.20			−353303.96	
B-N-O M2	11.32	2.94	8.04	1.99	0.51	1.15	2.79	1.45	−353300.55	6.81
S-N-O M0	11.31	2.88	8.22	2.15	0.73	1.17			−356074.00	
S-N-O M2	11.31	2.97	8.22	2.06	0.72	1.14	1.51	0.34	−356073.52	0.96
Hm-Hp-O M0	11.18	0.94	8.50	1.99	0.83	0.43			−335891.18	
Hm-Hp-O M2	11.18	1.03	8.22	1.94	0.85	0.36	6.90	0.00	−335889.87	2.62
Hm-Hp-B M0	2.64	0.94	2.61	1.87	0.82	0.40			−129393.88	
Hm-Hp-B M2	2.62	1.34	2.66	1.42	0.76	0.32	6.28	16.46	−129390.74	6.28

Note.—The migration rate *M_ij_* = *N_j_m_ij_* is the expected number of immigrants in population *j* from population *i*.

For the Noncoding data, gene flow is detected at the 1% level (with critical value $\chi_{2,\;1\%}^{2}$ = 9.21) from the Hm-B-O, Hm-N-O, B-S-O, Hm-Hp-O, and Hm-Hp-B data sets. In the analysis of the two *Hylobates* species, use of different outgroups such as the human (O) and *Hoolock leuconedys* (B) produced consistent results. In both cases, migration from *H. moloch* to *H. pileatus* was inferred, at the rate of *M* = 0.0084 ± 0.0008 migrants per generation in the case of the human outgroup or 0.0078 ± 0.0012 for the *H. leuconedys* outgroup. In neither case was migration inferred in the opposite direction from *H. pileatus* to *H. moloch*. Migration between the two *Hylobates* species should have the effect of causing bpp (which ignores migration) to group the two species into the same clade, although the monophyly of the *Hylobates* genus was not in doubt. For all other species pairs, the migration rate was around 0.001 migrant individuals per generation or lower. Migration rates of such magnitude are expected to have little impact on species tree estimation (Leaché et al. 2014).

For the Coding data, the LRT did not reach the 1% level for any of the data sets, whereas at the 5% level (with critical value $\chi_{2,\;5\%}^{2}$ = 5.99), there was evidence for gene flow between species pairs Hm-B, B-S, B-N, and Hm-Hp (table 6). For the Hm-Hp pair, parameter estimates suggested a migration rate of 0.007 ± 0.003 from *H. moloch* to *H. pileatus*, and no migration in the reverse direction when the human was used as the outgroup, consistent with the analysis of the Noncoding data set. When *H. leuconedys* was used as the outgroup, the estimates were 0.006 ± 0.003 from Hm to Hp, and 0.016 ± 0.009 in the reverse direction from Hp to Hm. The large standard errors indicate that the estimates may be unreliable. As the results differed among replicate datasets and among different choices of the outgroup, and the test is only marginally significant, we do not emphasize the estimates from the Coding data. In general, the coding data are much less informative than the Noncoding data. For the other cross-genera species pairs, either the migration rates are low or the test is not highly significant.

We note that MLEs of parameters for the root of the species tree (*τ_ABC_* and *θ_ABC_*) are very similar between models M0 and M2 and also very similar to the Bayesian estimates from the full data (supplementary table S1, Supplementary Material online). For example, for the Noncoding data, the MLEs are ≈0.0151 for *τ_ABC_* and ≈0.0056 for *θ_ABC_*, compared with the Bayesian posterior means 0.0154 and 0.0055. For the Coding data, the MLEs are 0.0112–0.0113 for *τ_ABC_* and 0.0080–0.0082 for *θ_ABC_*, compared with the posterior means 0.0115 and 0.0078. Population size parameters (*θ*s) for the modern species are also extremely similar between the ML and Bayesian analyses.

Because the four gibbon genera diverged at very similar times, the age *τ_AB_* in the first six data sets (table 6) maps either exactly or approximately to the root of the gibbon clade in the species tree (fig. 1*A* and *B*). Its MLE was ∼0.0043 for the Noncoding data and ∼0.0028 for the Coding data, slightly smaller than the posterior means from the full data (0.0045 for Noncoding and 0.0031 for Coding). Parameter *θ_AB_* (with MLEs 0.0023–0.0025) mostly reflect the long branch ancestral to the gibbon clade in figure 1*A* and *B* (with posterior mean 0.0024 in the bpp analysis), because the internal branches inside the gibbon clade are all very short. Even though the 3s analysis used three sequences per locus, whereas the bpp analysis used 17, data sets of over 10,000 loci are informative enough for the two methods to produce highly similar parameter estimates.

When there is gene flow between species *A* and *B*, ignoring gene flow can lead to biased parameter estimates. Compared with estimates under M0 (no gene flow), M2 (gene flow) produced larger *τ_AB_* and smaller *θ_AB_* estimates. In other words, if the migration between *A* and *B* is ignored, one will underestimate the species divergence *τ_AB_* and overestimate the ancestral population size parameter *θ_AB_*. The size of the population receiving immigrants will also be seriously overestimated. In computer simulations, a small amount of migration was noted to affect parameter estimation more than species tree estimation (Leaché et al. 2014).

## Discussion

### Utility of Coding Sequences in Inference under the MSC

The coding loci are clearly under purifying selection, which removes deleterious nonsynonymous mutations, while the MSC model assumes neutral sequence evolution. However, we expect that protein-coding genes under Darwinian selection or species-specific directional selection are rare in the gibbon genome and that most genes or exons are performing the same functions and are under similar selective constraints among the gibbon species. Purifying selection thus has the predominant role of reducing the neutral mutation rate, with a less important role of affecting the shape of the gene trees. We thus suggest that coding loci may be sensibly analyzed under the MSC. Indeed in this study the coding and noncoding data sets produced highly consistent results in terms of both the species tree topology and the parameters in the MSC model, highlighting the utility of examining different parts of the genome for such analyses (see also Ebersberger et al. 2007; Dalquen et al. 2017).

As mentioned earlier, the posterior means of *τ*s (which measure the between-species divergences) and *θ*s (which measure the within-species polymorphism) form near perfect linear relationships between the coding and noncoding data sets, with *τ*_(C)_ = 0.73*τ*_(NC)_ and *θ*_(C)_ = 0.62*θ*_(NC)_. If the noncoding loci and the synonymous sites in the coding exons are evolving neutrally and if the proportion of synonymous sites in the exons is 1/4, then the slope of 0.73 may be translated into an average genome-wide estimate of the nonsynonymous/synonymous rate ratio of *ω* = 0.64 (since 1/4+3/4 ω = 0.73).

We suggest that the smaller slope for *θ* than for *τ* (0.62 vs. 0.73) is expected from the population genetics theory of background selection, which predicts a reduction in polymorphism at a neutral locus due to its linkage to sites or loci under purifying selection (Charlesworth et al. 1993; Hudson and Kaplan 1995; Nordborg et al. 1996; McVicker et al. 2009). Recall that both *τ* and *θ* are defined on a per-site basis. Suppose we use a particular site in the exon as reference to define *τ* and *θ*, and assume no recombination within the exon so that all sites in the exon share the same genealogical history. As a simple model, assume that mutations in the noncoding loci are neutral and mutations in the exon consist of three types: neutral synonymous mutations, and lethal and deleterious nonsynonymous mutations. We first consider mutations at the reference site and then the impact of selection on sites elsewhere in the exon. Neutral mutations at the reference site are fixed at the same rate as mutations in the noncoding loci even though they are linked to nonsynonymous sites under purifying selection elsewhere in the exon (Birky and Walsh 1998). Lethal nonsynonymous mutations have the effect of reducing the neutral mutation rate. Those two types of mutations at the reference site have the same effect on divergence and polymorphism (*τ* and *θ*). Slightly deleterious mutations at the reference site reduce the probability of fixation relative to neutral mutations and lead to a reduction in both divergence and polymorphism; this reduction may not be very different on *τ* and *θ*. However, *τ* and *θ* (defined for the reference site) are affected differently by selection removing lethal or deleterious mutations elsewhere in the exon. When a lethal or deleterious mutation at any other site in the exon is removed in the population, the linked allele at the reference site will be lost. Such background selection will cause a reduction in the effective population size or the average coalescent time between alleles at the reference site (*θ*), but have no effect on divergence (*τ*). The effect will depend on the combined strength of purifying selection across all codons in the exon and may be greater in a longer exon if selective strength is comparable on a per-codon basis. At any rate, background selection should be the main factor accounting for the smaller slope for *θ* than for *τ* observed in the gibbon data. The model we consider here is simplistic and does not account for variable selection among different genes or exons or among different sites of the same exon. It will be interesting to explore the potential of using the MSC framework to study the distribution of selective coefficients of nonsynonymous mutations in the genome.

We note that background selection, especially when selection is weak, can distort the shape of the gene genealogy, resulting in longer external branches in the gene tree or an excess of rare variants relative to the neutral expectation (Charlesworth et al. 1993; Fu 1997; Zeng and Charlesworth 2011). This constitutes a violation of the assumption of the MSC model that mutations do not affect the gene tree distribution (Rannala and Yang 2003). The model violation may be expected to have a larger effect on the estimation of *θ*s, as discussed above, than on the species tree topology. Here, we emphasize the fact that the two sets of loci, although under very different selective pressures, produced consistent estimates of the species tree topology and parameters, with a neutral mutation rate difference of ∼73%.

### Computational Challenges of Bayesian MSC Methods

Full-likelihood implementations of the MSC model as in bpp involve intensive computation. While computation increases with the increase in the number of species, the number of loci, the number of sequences at each locus, and the number of sites in the sequence, the most important factor appears to be the increased difficulty of moving from one species tree to another when a large number of loci are analyzed (Rannala and Yang 2017). The six full data sets analyzed in this study, each with ≥10,000 loci, are unprecedented for Bayesian species tree estimation. Indeed we observed mixing problems with bpp in three of them, with the Markov chain getting stuck in tree 2 even though the MAP tree (tree 1) is a simple NNI-move away.

While there are five gibbon species in the data analyzed in this study, there is never uncertainty concerning the two *Hylobates* species grouping into one clade: effectively only the 15 possible species trees for the four genera (table 1) were entertained in all analyses. We note that in the difficult data sets where bpp had mixing problems, the chain was able to move freely between species trees during the early stages of the run, but sometimes became stuck in tree 2 at later stages of the run. There did not appear to be any relation between the starting species tree and the final tree (either tree 1 or tree 2) that the chain eventually settled in. The mixing difficulty appears to be due to the fact that the gene trees (topologies and branch lengths) are nearly optimized for the sequence data (within the constraint of species tree 2), and that when the algorithm attempts to move from species tree 2 to tree 1, the new gene trees—generated in the proposal by applying a number of SPR manipulations on the current gene trees (Yang and Rannala 2014; Rannala and Yang 2017)—tend to be poor, leading to the rejection of the proposed species tree 1 even though it has overall a higher posterior than species tree 2. We hope to develop smart MCMC proposal algorithms by generating better gene trees to improve the acceptance rate of such moves across species trees. Our results also highlight the importance of running the same analysis multiple times as a means of diagnosing mixing problems with transmodel MCMC algorithms.

### Concatenation and the Anomaly Zone

Analyses of simulated data sets allowed us to compare the statistical efficiency of the methods used in this study. Our simulation was used to aid the interpretation of the real data analysis, and is not intended to be a comprehensive simulation study. In particular, we did not explore the parameter space extensively and considered only challenging shallow species trees characterized by extremely short internal branches with data consisting of many loci of weak phylogenetic information.

To “quantify” the challenge of the gibbon species tree, we used MCcoal to simulate the gene-tree distribution under the MSC model, using parameter estimates for the coding and noncoding data of figure 1*A* and *B*. This is the same simulation as discussed above except that we use one sequence per species. The gene-tree distribution can be calculated analytically using the algorithm of Degnan and Rosenberg (2006) but here we use MCcoal to simulate 10^7^ (true) gene trees. For the noncoding (or coding) data, the majority-rule consensus tree of all simulated gene trees has only two resolved nodes, the H node (for Hm–Hp) with frequency 92.0% (or 89.5% for coding) and the gibbon node (exclusive of the human outgroup) with frequency 100.0% (or 100.0%); no other nodes occur in more than half of the gene trees. Gene tree 1, (((B, S), N), H), which matches the species tree, has frequency 8.3% (or 8.2% for coding loci) so that for ∼92% of the genome, the gene tree has different topologies from the species tree. With so much incomplete lineage sorting and gene tree fluctuations across the genome, the gibbon phylogeny is indeed a hard problem. The most common gene tree is tree 7, ((B, S), (H, N)), with frequency 11.3% (or 11.0%). Thus a majority-vote approach, which uses the most common gene tree as the estimate of the species tree, will be inconsistent, and the species tree is in the *anomaly zone* (Degnan and Rosenberg 2006).

Indeed this case of an unbalanced species tree for four species is the simplest case for anomalous gene trees (Degnan and Salter 2005; Yang 2014, p. 333–5). When the true species tree is tree 1 with two extremely short internal branches, most coalescent events occur in the common ancestor that is the root of the species tree. Then the matching gene tree 1 occurs with probability ∼1/18 while the mismatching gene tree 7, ((B, S), (H, N)), has probability ∼2/18, because tree 1 can arise through only one sequence of coalescent events (B-S followed by BS-N) but tree 7 can arise through two (either B-S followed by H-N or H-N followed by B-S). In other words, the coalescent process assigns equal probabilities to labeled histories (which are rooted gene trees with internal nodes ordered by age) but not to rooted gene trees. If the internal branches in the true species tree 1 are sufficiently short, it will be possible for tree 7 to have a greater probability than tree 1 (although not twice as great). In such a case, majority vote will be inconsistent; it will produce the wrong species tree 7 with higher probability, the more genes or gene trees are in the data. The argument here assumes true gene trees. In real data analysis, phylogenetic errors will alter the gene tree probabilities, so that the boundaries of the anomaly zone will be more complex (Yang 2002; Huang and Knowles 2009).

Similar to majority vote, concatenation is known to produce strongly supported but incorrect species trees when the internal branches in the species tree are very short (Giarla and Esselstyn 2015). It has anomaly zones similar to majority-vote, although the boundaries are different (Kubatko and Degnan 2007; Roch and Steel 2015). The results of table 4 suggest that the species trees of figure 1*A* and *B* may be in the anomaly zone for concatenation. Our results support the early suggestion that concatenation is not suitable for challenging species tree problems (Giarla and Esselstyn 2015; Kubatko and Degnan 2007; Edwards et al. 2016).

We also note that in the concatenation analysis ML under GTR+Γ_4_ performed in general worse than under JC. Judged by the log-likelihood values, GTR+Γ_4_ fits the JC data much better than JC, with Δℓ = 42,133 between the two models for NoncodingJC and 6,398 for CodingJC. The difference is even much greater for the GTR data sets. Use of any model-selection criterion will lead to rejection of JC by a huge margin. However, in this case the fault lies with concatenation fitting one tree with branch lengths to all loci and all sites in the data set, and not with the assumed model of nucleotide substitution. The GTR+Γ_4_ model misinterprets the heterogeneity among loci in the gene tree topologies and coalescent times, which is predicted by the coalescent theory (Rannala and Yang 2003), as substitution rate heterogeneity among sites in the concatenated alignment. Our results highlight the importance of considering model robustness or the impact of model assumptions on the analysis, and argue against the mechanical use of model-selection procedures (such as the LRT, AIC, BIC, and Bayes factor) that appear to be common in modern phylogenetic analysis.

### The Assumptions and Performance of SVDquartets

While concatenation performed poorly on the simulated data, the coalescent-based species tree methods also showed large performance differences in the simulated data subsets. In general, bpp performed better than astral, whereas SVDQuartets was the worst (tables 2 and 3). The poorer performance of astral and in particular SVDquartets than bpp on the GTR data (table 3) may seem surprising because the astral/PhyML analysis assumed GTR+Γ and SVDquartets assumes GTR+Γ+I so that all model assumptions are satisfied for both methods, whereas bpp assumes JC, which is seriously violated. We note that the performance difference of table 3 is not due to the small number of simulation replicates. Figure 6 shows a similar simulation with different numbers of loci. A large performance gap exists between the full likelihood method (bpp) and the summary methods (astral, SVDQuartets). Whereas bpp was able to infer the true tree with high accuracy with 500 loci (99% for noncoding and 71% for coding), SVDQuartets had little power at this data size (36% and 28%).

Here, we discuss two factors that may account for the poor performance of SVDquartets in our simulations. We focus on SVDQuartets as it produced a different species tree (tree 7) from the real full data sets than bpp and astral. We believe that the quartet-assembly algorithm is not to blame, and focus here on the case of four species and four sequences, with one sequence from each species. First, SVDquartets is a heuristic method based on summary statistics and its use of data summaries instead of the full likelihood leads to unidentifiability of model parameters and loss of power in species tree estimation. The method does not operate on sequence alignments, and instead merges all sites across all loci to generate the counts of the 256 site patterns for four sequences, which are a marginal summary of the original sequence alignments at multiple loci. In the MSC, sites in the alignment for the same locus share the gene tree (topology and branch lengths), and analysis of sequence alignments at multiple loci allows full likelihood methods such as bpp to tease apart the variation among sites of the same locus due to the Poisson mutation process and the variation among the gene trees for loci due to the coalescent process. Note that fluctuations in genealogical histories among loci provide important information about the coalescent process such as the ancestral population sizes. Merging sites across loci means that such information is lost and the two sources of variation are confounded. This summary of data leads to unidentifiability of parameters in the MSC model. For example, in the case of two species and two sequences, there are two parameters in the model (*θ* for the common ancestor and *τ* for the divergence time between the two species), but the summary data consist of only one observation, the proportion of different sites, so that it is impossible to identify two parameters. (Here, we use the JC model although the assumed mutation model is inconsequential to the argument.) Similar unidentifiability arises in the case of three species and three sequences, and the case of four species and four sequences. The method is thus able to identify the species tree topology (Chifman and Kubatko 2015), but not all parameters of the MSC model. This situation is similar to that for astral, which uses the gene tree topologies as data summary and is able to identify the species tree topology but not all parameters of the model (table 5). As in the case of astral, a loss of power in the estimation of the species tree topology due to the use of data summary is thus expected. It is also noteworthy that SVDquartets has similarity to concatenation in that both merge sites across loci. Both inferred the incorrect balanced species tree 7 in many of the simulated data subsets (table 2). Nevertheless, SVDquartets is a coalescent-based method, aware of the distinction between labeled histories and rooted trees so that it is consistent while concatenation may be inconsistent. The reasons why SVDquartets favored tree 7 are thus not so clear.

The decision to merge sites across loci appears to have been motivated by the discussion of Gatesy and Springer (2014) of the so-called coalescent-gene or *c*-gene, a gene locus without recombination throughout the gene tree. Springer and Gatesy (2016) calculated the *c*-gene size to be very short (∼12 bp for mammals, say), but that calculation may be too stringent, because recombination is relevant only during the gene history when there are two or more lineages so that it is possible for recombination to occur (Lanier and Knowles 2012; Edwards et al. 2016; Xu and Yang 2016). At any rate, in simulations SVDquartets failed to show an advantage over astral for very short alignments (Chou et al. 2015).

The second factor that may be important for the poor performance of SVDquartets is its departure from the principles of statistical inference and its reliance on phylogenetic invariance for tree comparison, which may lead to inefficiency and sensitivity to the details of the mutation model. In bpp and astral (PhyML), the mutation model is used to correct for multiple hits to estimate the gene tree topology and branch lengths, and the analysis is expected to be insensitive to the mutation model at high sequence similarity (e.g., Xu and Yang 2016). Note that the sequence distance between gibbons and humans is ∼3.6% at the noncoding loci and ∼3.0% at the coding loci, whereas between gibbons it is only 1.1% and 0.8% (table 7). At such divergence levels, any mutation model will produce a distance that is close to the raw proportion of differences (the so-called *p* distance). SVDquartets does not use information in the gene tree topologies or branch lengths, and instead rely on phylogenetic invariants. Given the data of 256 site pattern frequencies, the standard practice is to apply ML or BI to evaluate different species trees, as outlined by Xu and Yang (2016). Instead SVDquartets takes a mathematical shortcut. The expected site pattern probabilities, when arranged into a 16 × 16 matrix according to the true species tree, has rank ≤10, whereas the rank is >10 if the matrix is arranged according to an incorrect species tree. The rank of a square matrix is equal to the number of nonzero eigenvalues. A nonsingular 16 × 16 matrix has rank 16, but linear relationships among rows or columns reduce its rank. In other words, the site pattern probabilities generated by a species tree satisfy a number of linear relationships, depending on the assumed mutation model. The method uses a heuristic criterion to measure how close the 11–16th eigenvalues are to 0 (Chifman and Kubatko 2014, eq. 2). This departure from statistical principles (in particular, the likelihood principle) means that the method may be inefficient (Edwards 1972; Xu and Yang 2016). Its reliance on the symmetry relationships implied by the substitution model may mean high sensitivity to model assumptions (such as the detailed-balance condition of the GTR model). Even though both the GTR (Yang 1994a) and the Γ (Yang 1994b) components of the model are useful improvements to phylogenetic substitution models, they are never supposed to represent the truth when applied to real data. Complex features of the mutation process such as nonreversibility and context-dependence (Hwang and Green 2004) should have little effect on correction for multiple hits or on the performance of bpp when the sequences are highly similar, but they may well cause the symmetry conditions required by SVDquartets to break down. We leave it to future work to investigate which of these or other factors are the most important for the poor performance of SVDquartets.

**Table 7.** Average JC Distances across the Noncoding (lower triangle) and Coding (upper triangle) Loci, with the within-Species Distances on the Diagonal.

	B	S	N	Hm	Hp	Human
B	0.0009/0.0006	0.0076	0.0076	0.0078	0.0079	0.0307
S	0.0108	0.0012/0.0008	0.0079	0.0080	0.0081	0.0310
N	0.0108	0.0110	0.0018/0.0012	0.0080	0.0081	0.0307
Hm	0.0110	0.0111	0.0110	0.0014/0.0010	0.0039	0.0308
Hp	0.0111	0.0112	0.0111	0.0056	0.0006/0.0004	0.0309
Human	0.0359	0.0362	0.0358	0.0360	0.0361	NA

We note that our results concerning SVDQuartets are consistent with previous simulation studies that evaluated the method. Chifman and Kubatko (2014) simulated data using a balanced 4-species tree with equal internal and external branch lengths and found that SVDQuartets behaved well. Species trees of that shape are easy to recover as the internal branches are relatively long. In another simulation study, Chou et al. (2015) found that SVDquartets performed well when the species tree had long internal branches and incomplete lineage sorting was infrequent, but was inferior to astral when the species tree had very short internal branches and incomplete lineage sorting was common. In our simulation, the species tree (tree 1) had extremely short internal branches and accordingly SVDquartets performed poorly.

In summary, SVDQuartets made many errors in the simulated data subsets, where the incorrect inferred species tree was predominantly tree 7. The support values calculated by the method were unreliable and overconfident. By considering the construction of the method, we suggest that the method may be sensitive to details of the substitution model although this claim needs further verification. Similarly, its tendency to favor the balanced species tree 7 over the unbalanced species tree 1, as does concatenation in the anomaly zone, needs further investigation.

### Estimation of Gibbon Phylogeny as an Exemplar for Challenging Species Tree Problems

We examine some of the assumptions made in our analyses before reaching a conclusion concerning the gibbon phylogeny. First, we assumed the JC mutation/substitution model. The JC model is grossly wrong in terms of its fit to data. However, for closely related species like gibbons, JC should be adequate because the role of the model in bpp is to correct for multiple hits at the same site but multiple hits are rare between highly similar sequences (Yang 2015; Rannala and Yang 2017). The bpp analysis under JC of the data sets simulated under GTR+Γ confirmed this expectation (tables 2–4). Previously even the infinite-sites model produced very similar results to finite-sites models such as JC in analysis of data from the apes (Satta et al. 2004). Second, our bpp analyses assumed the molecular clock. The clock assumption was examined by Burgess and Yang (2008) in their analysis of the hominoid genomic sequence data, who found that the clock approximately holds and accommodating its violation had virtually no effect on estimation of parameters under the MSC model. Given that the gibbon species are even more closely related, we expect the clock to be adequate (see also table 7). Note that neither astral nor SVDQuartets assumes the clock and both use the human outgroup to root the trees, so that the different species trees inferred by the two methods in the two real full data sets cannot be explained by the assumption of the clock. Third, the species tree methods we used assume no migration or introgression. While introgression is a major complicating factor in many shallow phylogenies, it does not appear to be a serious issue for the gibbon data sets analyzed here. We have focused on the genus-level relationships so that the species involved are quite distant, and do not appear to hybridize today. Our analysis testing for migration and estimating the migration rates suggests possible gene flow from *H. moloch* to *H. pileatus* (at the low rate of ∼0.008 migrants per generation), which should have little effect on species tree estimation, while migration across genera is either absent or extremely low. We note that the test of Carbone et al. (2014) using the D-statistic (Durand et al. 2011) failed to identify unequivocal evidence of gene flow. In summary, we suggest that our species tree estimation may not have been affected by those simplistic assumptions.

Our simulation mimicking the real data sets has supported the reliability of bpp and astral, which were able to recover the true species tree despite the extremely short internal branches and widespread incomplete lineage sorting. The consistency of results between the coding and noncoding loci (despite their great differences in the selective pressure) is also indicative of the reliability the inferred species tree. Our results for the full data sets are consistent with the analysis of the data subsets, in which there is no mixing problem and only trees 1 and 2 received substantial support. Our results are also largely consistent with the sliding window analysis of Carbone et al. (2014), which slides 100-kb nonoverlapping windows along the genome, instead of the well-spaced short fragments analyzed in this paper. Species trees 1 and 2 were the top UPGMA gene trees found in 15.4% and 13.2% of the 100-kb windows (Carbone et al. 2014, supplementary table ST 8.4, Supplementary Material online). If 10-kb windows were used instead, trees 2 and 1 were the most frequent gene trees, with frequency 9.105% and 9.103% (Carbone et al. 2014, SI text 8.3). Thus even though the choice of the window size was arbitrary, there was a consistent signal of weak support for trees 1 and 2, whereas tree 7 ranked #7, found in only 5.6% of the 100-kb sliding windows. It was not found in any of the data subsets by bpp or astral (table 2). Tree 1 was also the NJ tree based on sequence divergences calculated over the whole genome (similar to *p* distance; Carbone et al. 2014).

Given the overall reliability of bpp and astral in the simulations, and the consistency of our results between the coding and noncoding data sets and with previous genome-scale analyses using sliding windows and genomic distances, we suggest that species tree 1 represents the true gibbon phylogeny, and that species tree 7, inferred by SVDQuartets, may be an artefact of the method.

Does the overall consistency of our results with the analyses of Carbone et al. (2014) mean that we merely confirm the result of Carbone et al. except for attaching a higher confidence? The answer to this question is “No”. The phylogenetic methods used by Carbone et al. (2014) are not based on the coalescent and fail to account for the gene tree heterogeneity across the genome. They are known to fail in challenging species tree problems characterized by short internal branches. The sliding window analysis produced results that depend on the window size, with the most frequent gene tree to be tree 1 for a window size of 100-kb and tree 2 for 10-kb, while in both cases the support is extremely low. The main conclusion from the analyses of Carbone et al. (2014; see also Veeramah et al. 2015) was that the gibbon species tree was a hard problem: even the existence of a binary tree for the gibbons was questioned. In contrast, full likelihood methods such as bpp can recover the true species tree with high probability and high confidence, as demonstrated by our simulations. To such methods, the heterogeneous gene trees across the genome are not really in conflict with the species tree, but are a natural outcome of the biological process of reproduction and random drift; they are not a curse but an important source of information for estimating evolutionary parameters such as ancestral population sizes and species divergence times.

The gibbons arose through a series of radiative speciation events, leading to nearly simultaneous divergences and an extremely hard species tree estimation problem. Here the bpp and astral analyses of the genome-scale data sets under the MSC model led to a fully resolved species tree for the five species or four genera of gibbons. Both independent data sets, Noncoding and Coding, strongly support the genus-level phylogeny: ((*Nomascus*, (*Hoolock*, *Symphalangus*)), *Hylobates*), with *Hylobates* to be the earliest diverging lineage. The knowledge of the gibbon species tree should be useful for a reinterpretation of the morphological, anatomical, and behavioral data. We leave such work for the future. Here we highlight the intriguing fact that at over 90% of the exonic loci, just like the noncoding DNA, the genes have different histories from the species phylogeny. It may be interesting to examine the posterior distribution of the gene trees for the individual exons, and to correlate the most likely gene tree with the evolution of the morphological characters or biological functions that are encoded by the exon.

Adaptive radiations create challenging species tree problems (Schluter 2000). However, the availability of genome-scale data sets and the development of powerful statistical inference methods offer hopes for their resolution. The coding loci analyzed in this study contain no or very few parsimony-informative sites, and gene trees inferred at such loci are highly uncertain. However, with thousands of loci, coalescent methods such as bpp and astral inferred the species tree with near certainty and our simulations support the reliability of such inference. Thus a reliable estimation of the species tree is possible even if the phylogenetic information at every locus is very weak and all gene trees are poor. These results run counter to the intuition that species trees can be only as good as the gene trees on which they are built (Salichos and Rokas 2013; Liu et al. 2015). We suggest that other canonical examples of recent adaptive radiations, such as Darwin’s finches on the Galápagos Islands (Petren et al. 2005), Cichlid fish in the African lakes (Salzburger et al. 2002), the Hawaiian honeycreepers (Lerner Heather et al. 2011), the Hawaiian silverswords (Baldwin and Sanderson 1998), and the Anolis lizards in Central and South America (Losos 2009), may be similarly resolved.

Adaptive radiations in deep phylogenies offer even greater challenges. With divergent sequences, multiple-hit correction becomes important, and the molecular clock is often violated. Extending the mutation/substitution model and relaxing the molecular clock will be important avenues for expanding the functionality of the bpp program. It should be straightforward to implement a complex substitution model such as GTR+Г instead of JC to correct for multiple hits, and it appears straightforward to modify the relaxed-clock models for analysis of mixed within- and between-species data (Xu and Yang 2016). However, the violation of the molecular clock means that, even if the rate drift is adequately accommodated in the model, the temporal information in the sequence data about the relative node ages in the gene trees may be seriously eroded. One may work with either rooted gene trees with node ages relying on relaxed-clock models or with unrooted gene tree topologies discarding branch-length information. It will be interesting to examine to what extent Bayesian full-likelihood methods are advantageous over heuristic methods that rely on gene tree topologies only when the molecular clock is seriously violated.

## Materials and Methods

### Gibbon Data Sets

We used two genome-scale data sets generated and analyzed previously by Carbone et al. (2014) and Veeramah et al. (2015). The first data set includes 12,413 loci, each of 1,000 bp in length, which are at least 50 kbp away from the nearest exons. This is referred to as the Noncoding data set. The second data set, referred to as the Coding data set, consists of 11,323 loci, each of 200 bp, which are exons or overlap with exons. One species, with two individuals, was sampled from each of the three genera: *Hoolock* (*H. leuconedys*), *Nomascus* (*N. leucogenys*), and *Symphalangus* (*S. syndactylus*). Two species, with one individual from each, were sampled from the fourth genus *Hylobates* (*H. moloch* and *H. pileatus*). Two phased sequences were included for every individual at every locus. A human genome (hg19) was included as the outgroup. Thus the alignment at every locus consists of 17 sequences. The number of parsimony-informative sites ranges from 5 to 78 (with a median of 23) among the noncoding loci, and from 0 to 18 (median 3) among the coding loci. All loci including those with no parsimony-informative sites were used; in the Bayesian analysis, these loci are informative about the population size parameters (*θ*s) and indirectly about the species tree.

As the bpp program involves intensive computation and may suffer from mixing problems in large data sets, we separated the noncoding loci into 24 smaller subsets according to their genomic locations in *N. leucogenys*. Each subset consisted of 500 loci whereas the last one had 913. Similarly, the coding loci were separated into 11 data subsets, each of 1,000 loci (1,323 for the last). Those are referred to as the Noncoding500 and Coding1000 data subsets, respectively. Those subsets as well as the two full data sets were analyzed using a variety of methods, including bpp (Yang 2015), astral (Mirarab and Warnow 2015), mp-est (Liu et al. 2010), SVDQuartets (Chifman and Kubatko 2014), and concatenation. The analysis of the data subsets allows us to evaluate the efficiency of the different species tree estimation methods and to assess possible heterogeneity in the evolutionary history across the genome.

### Estimation of Species Tree Using bpp

We used the Bayesian program bpp 3.3 (Rannala and Yang 2003, 2017; Yang and Rannala 2014) to infer the species tree and to estimate the parameters under the MSC model. Species assignment and delimitation were fixed (this is analysis A01 of Yang 2015). Gamma priors were assigned to the parameters, which are the species divergence times (*τ*s) and population size parameters (*θ*s), both of which are measured by the expected number of mutations or substitutions per site. For the noncoding data, we used *θ* ∼ G(2, 1,000), with mean 0.002, and *τ*_0_ ∼ G(1.6, 100), with mean 0.016, for the age of the root. For the coding data, we used *θ* ∼ G(2, 2,000) and *τ*_0_ ∼ G(2, 200). The shape parameter of the gamma distribution (*α* = 2) means that those priors are fairly diffuse, whereas the rate parameter (*β*) was chosen so that the prior mean (*α*/*β*) was reasonable. The sequence likelihood was calculated under the JC model (Jukes and Cantor 1969).

For each data set we conducted 10 independent runs, using different starting species trees. The burn-in was set to 10^5^ for all analyses except for the Noncoding500 data sets for which 2 × 10^5^ were used. We sampled 2 × 10^4^ trees after the burn-in with a sampling frequency of 10. MCMC convergence was assessed mainly through consistency of results between runs (Rannala and Yang 2017). When convergence was achieved, the samples were combined to generate the maximum *a posteriori* (MAP) species tree (i.e., the species tree with the highest posterior probability).

We also estimated the parameters of the MSC model with the species tree fixed at tree 1 (the A00 analysis, Yang 2015). We conducted 10 independent runs. The within-model MCMC algorithms in bpp use automatic step-length adjustments and have good mixing efficiencies.

Computing time for each run on a single core was ∼10 h for each Noncoding500 data set, ∼80 h for Coding1000, and ∼200 h for the full data sets: Noncoding and Coding.

### Estimation of Species Tree Using astral, mp-est, and SVDquartets

We used astral 4.10.8 to estimate the species tree topologies and internal branch lengths and to calculate local posterior probabilities (Mirarab et al. 2014; Mirarab and Warnow 2015; Sayyari and Mirarab 2016). astral is a coalescent-based gene-tree summary method that operates on quartets. It collects the quartet trees in all the reconstructed unrooted gene trees, and evaluates different species trees according to how well they match the quartet trees in the set (Mirarab et al. 2014). We used the ML method implemented in PhyML v3 (Guindon et al. 2010) to reconstruct unrooted gene trees under the JC model. Short branch lengths in the gene trees (<10^−6^) were collapsed into polytomies using the *di2multi* function in ape (Paradis et al. 2004), before the gene trees were processed by astral. The ML gene tree should be preferred over bootstrapped gene trees as the former is more likely to match the true gene tree (Xu and Yang 2016): for example, Mirarab et al. (2016) found that use of bootstrapped gene trees led to deteriorated performance by astral.

We also inferred species trees from the ML gene trees using mp-est 1.5 (Liu et al. 2010). The mp-est method estimates species tree from a set of rooted gene trees by maximizing a pseudo-likelihood, which is the probability of the three alternative gene trees given a triplet species tree. Gene trees reconstructed using PhyML were rooted with the outgroup (human) before they were used by mp-est to estimate the species tree. We ran 100 independent searches for the maximum pseudo-likelihood tree.

SVDquartets (for Singular Value Decomposition for quartets) is another quartet-based summary method (Chifman and Kubatko 2014). For every quartet (four sequences from four species), the competing species trees are evaluated using a criterion based on phylogenetic invariant under the assumption that different sites in the sequence data have independent histories given the species tree (see Xu and Yang 2016 and Discussion of this paper). Like astral, the method infers an unrooted tree, with the outgroup (human) used to root the species tree. We used the implementation in paup* version 4.0a151 and evaluated all possible quartets. Node supports were calculated by using 1,000 bootstrap replicates.

### Estimation of Species Tree Using Concatenation

We applied concatenation analysis to the two full data sets: Noncoding and Coding. For each one, the sequences from the same individual were merged across loci to form a “supergene”. The resulting super-matrix of sequence alignment was analyzed using ML (PhyML, Guindon et al. 2010) and BI (MrBayes3.2.6, Ronquist et al. 2012), and the resulting ML tree or MAP tree was taken as the estimate of the species tree. Two nucleotide substitution models were used: JC (Jukes and Cantor 1969) and GTR+Γ_4_ (Yang 1994a, 1994b). Node supports on the ML tree were calculated by using 1,000 bootstrap replicates. MrBayes analysis used four chains (one cold and three hot), with the “temperature” parameter set to 0.2. The chain is started with random starting trees and run for 4 × 10^6^ iterations, sampling every 400 iterations. The MAP tree as well as the majority-rule consensus tree were generated using the sample from the cold chain, after the first 40% of the sample was discarded as burn-in.

Computation for astral, SVDquartets, and concatenation by ML was incomparably faster than for bpp. MrBayes had mixing problems for Noncoding (and the simulated counterpart, NoncodingJC and NoncodingGTR), as the data sets with >10^7^ sites are large. The program always converged to the same tree, but had trouble traversing the space of the branch lengths for the same topology, with different runs visiting different branch lengths and achieving different log-likelihood values.

### Simulation

Our species tree estimation analyses suggest that species tree 1 of figure 1*A* and *B* is the best estimate for both full data sets (Noncoding and Coding). We used species tree 1 and the parameter estimates under the MSC (the posterior means of *τ*s and *θ*s in the A00 analysis) to simulate two data sets under JC (Jukes and Cantor 1969) (NoncodingJC and CodingJC) and two data sets under GTR+Γ_5_ (Yang 1994a, 1994b) (NoncodingGTR and CodingGTR). The MCcoal program in bpp was used. The same taxa sampling scheme was used as in the real data. Each of the noncoding data sets (NoncodingJC and NoncodingGTR) includes 10,000 alignments (loci) each of 1,000 bp, whereas each of the coding data sets (CodingJC and CodingGTR) includes 10,000 loci each of 200 bp. The two GTR data sets were generated mainly to examine the robustness of bpp, which currently implements the JC model only. To allow for heterogeneous mutation processes among loci, we sample the substitution parameters for the GTR model for each locus. The base frequencies (*π_T_*, *π_C_*, *π_A_*, *π_G_*) are sampled from the Dirichlet distributions D(44.8, 30.5, 44.8, 30.6) for NoncodingGTR and D(11.7, 11.4, 11.7, 11.3) for CodingGTR. Those values are ML estimates when the Dirichlet distribution was fitted to the observed base frequencies in the real data sets. Most of the loci are not informative enough to estimate the other parameters of the GTR+Γ model, and we sample those parameters as follows. The exchangeability parameters (*a*, *b*, *c*, *d*, *e*, *f*; Yang 1994a) are sampled from D(10, 5, 5, 5, 5, 10) for both the coding and noncoding loci, with an expected transition/transversion rate ratio of *κ* ≈ 2, whereas the gamma shape parameter for rate variation among sites (Yang 1994b) is generated from the gamma distribution G(100, 20) with mean 5 for NoncodingGTR, and from G(100, 50) with mean 2 for CodingGTR. The molecular clock is assumed in the simulation.

The simulated data sets were subjected to the same analyses as the real data sets. The 10,000 noncoding loci were analyzed as a whole and then divided into 20 subsets of 500 loci each. The 10,000 coding loci were analyzed as a whole and then divided into 10 subsets of 1,000 loci each. The bpp analysis always assumed the JC model, with the same prior specifications as in the analysis of the real data. The PhyML analysis, used by astral, assumed the JC model for the real and the JC data sets, and GTR+Γ_4_ for the GTR data sets.

### Test of Migration and Estimation of Migration Rates

Dealing with both incomplete lineage sorting and migration or hybridization is challenging. We used the ML program 3s to test for gene flow between the gibbon species and to estimate the directions and rates of migration (Zhu and Yang 2012; Dalquen et al. 2017). This is a full likelihood implementation of the MSC model with migration (or the isolation-with-migration, IM, model, Hey 2010) that can handle thousands of loci. However, 3s is limited to three species with three sequences per locus. We thus constructed eight triplet data sets by sampling three sequences per locus from each of the Coding and Noncoding data sets (table 6). The first six triplets are for testing gene flow between any pair of the four gibbon genera, with *Hylobates* represented by *H. moloch* (Hm). Two more data sets are for testing gene flow between the two *Hylobates* species, with either human (O) or *H. leuconedys* (B) as the outgroup.

Let the species tree be ((*A*, *B*), *C*), in which *A* and *B* are the ingroup species with possible gene flow whereas *C* is the outgroup involving no gene flow. Three sequences were sampled at random at each locus, with half of the loci having the configuration *ABC* (meaning one sequence from each species), a quarter of *AAC* (two sequences from *A* and one from *C*) and another quarter of *BBC*. The data were analyzed under two models. Model M0 (no gene flow) assumes no migration and involve six parameters: *τ_ABC_*, *τ_AB_*, *θ_ABC_*, *θ_AB_*, *θ_A_*, and *θ_B_*, whereas model M2 (gene flow) allows migration between *A* and *B*, with two additional migration rate parameters: *M_AB_* and *M_BA_*, where *M_ij_* = *N_j_m_ij_* is the expected number of immigrants in population *j* from population *i* per generation. The likelihood function for the sequence data is calculated by summing over the gene tree topologies and integrating over the two coalescent times by Gaussian–Legendre quadrature, using 32 points (Yang 2002; Dalquen et al. 2017). Gene flow between species *A* and *B* is tested using an LRT comparing models M0 and M2, using the *χ*^2^ distribution with df = 2. ML iteration to fit the two models to each data set took ∼5 min on an IBM Intel Xeon server with 80 cores.

## Supplementary Material

Supplementary data are available at *Molecular Biology and Evolution* online.

## Supplementary Material

Supplementary Data
