# Supplementary material for: Coalescent-Based Analyses of Genomic Sequence Data Provide a Robust Resolution of Phylogenetic Relationships among Major Groups of Gibbons
Source: Mol Biol Evol. 2017 Oct 25;35(1):159–79. doi: 10.1093/molbev/msx277 (PMC5850733; doi:10.1093/molbev/msx277)
Supplement: Supplementary Data [file msx277_supp.zip › TableS1TreeI_ThetaTau.pdf]

Supplemental Table S1. Parameter estimates for the two real and four simulated full datasets from the BPP A00 analysis (with the species tree 1 fixed)

NoncodingAll

	theta_1Hm	theta_2Hp	theta_3B	theta_4S	theta_5N	theta_7ONBSHmHp	theta_8NBSHmHp	theta_9NBS	theta_10BS	theta_11HmHp	tau_7ONBSHmHp	tau_8NBSHmHp	tau_9NBS	tau_10BS	tau_11HmHp
mean	0.001297	0.000609	0.000943	0.001278	0.001941	0.005501	0.002432	0.016392	0.006667	0.002511	0.015338	0.004494	0.003854	0.003637	0.001569
2.5%HPD	0.001267	0.000593	0.000927	0.001258	0.001913	0.005309	0.00238	0.014241	0.00233	0.002437	0.015242	0.004471	0.003794	0.003521	0.001541
97.5%HPD	0.001329	0.000626	0.000959	0.001298	0.001968	0.005691	0.002482	0.018632	0.010892	0.002582	0.015425	0.004518	0.003914	0.003759	0.001596
ESS*	134344.1	16184.6	9702.8	30316.3	92715.6	11974.7	57246.8	850.9	258.1	108508.6	10908.8	70519.1	1172.2	309	78418.7
Eff*	0.671721	0.080923	0.048514	0.151581	0.463578	0.059874	0.286234	0.004254	0.001291	0.542543	0.054544	0.352596	0.005861	0.001545	0.392093

CodingAll

	theta_1Hm	theta_2Hp	theta_3B	theta_4S	theta_5N	theta_7ONBSHmHp	theta_8NBSHmHp	theta_9NBS	theta_10BS	theta_11HmHp	tau_7ONBSHmHp	tau_8NBSHmHp	tau_9NBS	tau_10BS	tau_11HmHp
mean	0.000827	0.00043	0.000581	0.000751	0.001207	0.00784	0.001859	0.010362	0.026721	0.002123	0.011482	0.003061	0.002676	0.001724	0.000885
2.5%HPD	0.000786	0.000405	0.000558	0.000723	0.001172	0.007418	0.001785	0.005831	0.020837	0.002004	0.011272	0.003017	0.002524	0.001608	0.000838
97.5%HPD	0.000871	0.000454	0.000604	0.000778	0.001243	0.008257	0.001932	0.015468	0.032836	0.00224	0.011687	0.003103	0.002826	0.001842	0.000931
ESS*	9294.8	1495.1	767.1	1502.7	3483.2	1455.8	5464	210.3	157.9	9471.3	1409	9767.8	321.3	1457.1	5038.7
Eff*	0.048152	0.007746	0.003974	0.007785	0.018045	0.007542	0.028307	0.00109	0.000818	0.049066	0.007299	0.050603	0.001665	0.007548	0.026103
											1.335829995	1.468147664	1.4402093	2.1096288	1.772881356

NoncodingJC

	theta_1Hm	theta_2Hp	theta_3B	theta_4S	theta_5N	theta_7ONBSHmHp	theta_8NBSHmHp	theta_9NBS	theta_10BS	theta_11HmHp	tau_7ONBSHmHp	tau_8NBSHmHp	tau_9NBS	tau_10BS	tau_11HmHp
mean	0.001268	0.00061	0.000945	0.001297	0.001966	0.005523	0.002484	0.016308	0.004313	0.002596	0.015365	0.004483	0.003823	0.003685	0.001545
2.5%HPD	0.001235	0.000592	0.000928	0.001274	0.001935	0.005316	0.00243	0.013264	0.001111	0.002516	0.015266	0.004458	0.003746	0.003571	0.001515
97.5%HPD	0.001303	0.000629	0.000963	0.001319	0.001997	0.005729	0.002537	0.019558	0.008128	0.002675	0.015462	0.004507	0.003902	0.003789	0.001573
ESS*	134729.6	16453.5	11629.5	33831.6	29766.3	12529.2	23923.8	520.4	279.4	125893.1	11477.1	112275	669	440	87499.4
Eff*	0.700018	0.085488	0.060424	0.17578	0.154657	0.065098	0.124301	0.002704	0.001452	0.654106	0.059632	0.58335	0.003476	0.002286	0.454623

CodingJC

	theta_1Hm	theta_2Hp	theta_3B	theta_4S	theta_5N	theta_7ONBSHmHp	theta_8NBSHmHp	theta_9NBS	theta_10BS	theta_11HmHp	tau_7ONBSHmHp	tau_8NBSHmHp	tau_9NBS	tau_10BS	tau_11HmHp
mean	0.000839	0.000458	0.000605	0.000779	0.001227	0.0077	0.001937	0.003632	0.017049	0.002074	0.011421	0.003049	0.00287	0.001948	0.000911
2.5%HPD	0.000794	0.000431	0.00058	0.000749	0.001189	0.007269	0.001858	0.000978	0.012592	0.001951	0.011197	0.003004	0.002726	0.001795	0.000861
97.5%HPD	0.000885	0.000486	0.00063	0.00081	0.001264	0.008147	0.002014	0.006635	0.021428	0.0022	0.011637	0.003094	0.002997	0.00211	0.000961
ESS*	19092.7	4158	1113.8	1836.7	5297.1	1572.4	3206.2	114.7	184.8	14081.2	1544.3	3233.4	139	631.2	11913.3
Eff*	0.095464	0.02079	0.005569	0.009183	0.026486	0.007862	0.016031	0.000573	0.000924	0.070406	0.007721	0.016167	0.000695	0.003156	0.059566

NoncodingGTR

	theta_1Hm	theta_2Hp	theta_3B	theta_4S	theta_5N	theta_7ONBSHmHp	theta_8NBSHmHp	theta_9NBS	theta_10BS	theta_11HmHp	tau_7ONBSHmHp	tau_8NBSHmHp	tau_9NBS	tau_10BS	tau_11HmHp
mean	0.001251	0.0006	0.000931	0.001248	0.001909	0.01279	0.002716	0.017342	0.004575	0.002462	0.011542	0.004381	0.003789	0.00363	0.001528
2.5%HPD	0.001217	0.000582	0.000914	0.001226	0.001879	0.012458	0.002655	0.013774	0.000598	0.002383	0.011433	0.004354	0.003703	0.003513	0.001497
97.5%HPD	0.001284	0.000619	0.000949	0.001269	0.001939	0.013115	0.002778	0.021129	0.008218	0.002538	0.011651	0.004406	0.003874	0.003749	0.001557
ESS*	104238.1	9154.5	7078.4	16915.5	24383.7	9058.1	22630.5	502.2	272.4	120489	4317.5	90624.6	609	426.4	78597.2
Eff*	0.52119	0.045773	0.035392	0.084577	0.121919	0.045291	0.113153	0.002511	0.001362	0.602445	0.021587	0.453123	0.003045	0.002132	0.392986

CodingGTR

	theta_1Hm	theta_2Hp	theta_3B	theta_4S	theta_5N	theta_7ONBSHmHp	theta_8NBSHmHp	theta_9NBS	theta_10BS	theta_11HmHp	tau_7ONBSHmHp	tau_8NBSHmHp	tau_9NBS	tau_10BS	tau_11HmHp
mean	0.000796	0.000422	0.000591	0.000756	0.001202	0.014518	0.001901	0.003215	0.017467	0.002208	0.008047	0.00301	0.002892	0.001905	0.000836
2.5%HPD	0.000752	0.000397	0.000566	0.000726	0.001166	0.014051	0.001829	0.000844	0.013249	0.002086	0.007865	0.002965	0.002769	0.001749	0.00079
97.5%HPD	0.00084	0.000447	0.000616	0.000786	0.001239	0.014993	0.001976	0.006282	0.021892	0.002335	0.008234	0.003054	0.002991	0.002064	0.000884
ESS*	11688.5	1866	608.7	1433.4	7343	1296.1	3187.2	210.9	362.8	9666.1	808.7	3087.4	248.2	788.6	6739.8
Eff*	0.059941	0.009569	0.003121	0.007351	0.037656	0.006647	0.016345	0.001082	0.001861	0.04957	0.004147	0.015833	0.001273	0.004044	0.034563
